# Molecular characterization of emaraviruses associated with Pigeonpea sterility mosaic disease

**DOI:** 10.1038/s41598-017-11958-8

**Published:** 2017-09-19

**Authors:** Surender Kumar, BL Subbarao, Vipin Hallan

**Affiliations:** 1 Academy of Scientific and Innovative Research (AcSIR), CSIR-Institute of Himalayan Bioresource Technology (CSIR-IHBT) Campus, Palampur, 176061 India; 2 Plant Virology Lab, Biotechnology Division, CSIR-Institute of Himalayan Bioresource Technology, Palampur, HP 176061 India; 3 House # B-88, 3rd Ave, 6th Cross, Sainikpuri, Secunderabad, 500 094 Telangana India

## Abstract

Sterility Mosaic Disease (SMD) of pigeonpea (*Cajanus cajan* (L.) Millspaugh) is a complex disease due to various factors including the presence of a mixed infection. Comparison of dsRNA profile and small RNA (sRNA) deep sequencing analysis of samples from three locations revealed the presence of Pigeonpea sterility mosaic virus-I and II (PPSMV-I and II) from Chevella and only PPSMV-II from Bengaluru and Coimbatore. PPSMV-I genome consisted of four while PPSMV-II encompassed six RNAs. The two viruses have modest sequence homology between their corresponding RNA 1–4 encoding RdRp, glycoprotein precursor, nucleocapsid and movement proteins and the corresponding orthologs of other emaraviruses. However, PPSMV-II is more related to *Fig mosaic virus* (FMV) than to PPSMV-I. ELISA based detection methodology was standardized to identify these two viruses, uniquely. Mite inoculation of sub-isolate Chevella sometimes resulted in few- to- many pigeonpea plants containing PPSMV-I alone. The study shows that (i) the N-terminal region of RdRp (SRD-1) of both the viruses contain “cap-snatching” endonuclease domain and a 13 AA cap binding site at the C-terminal, essential for viral cap-dependent transcription similar to the members of *Bunyaviridae* family and (ii) P4 is the movement protein and may belong to ‘30 K superfamily’ of MPs.

## Introduction

**Figure 1:**
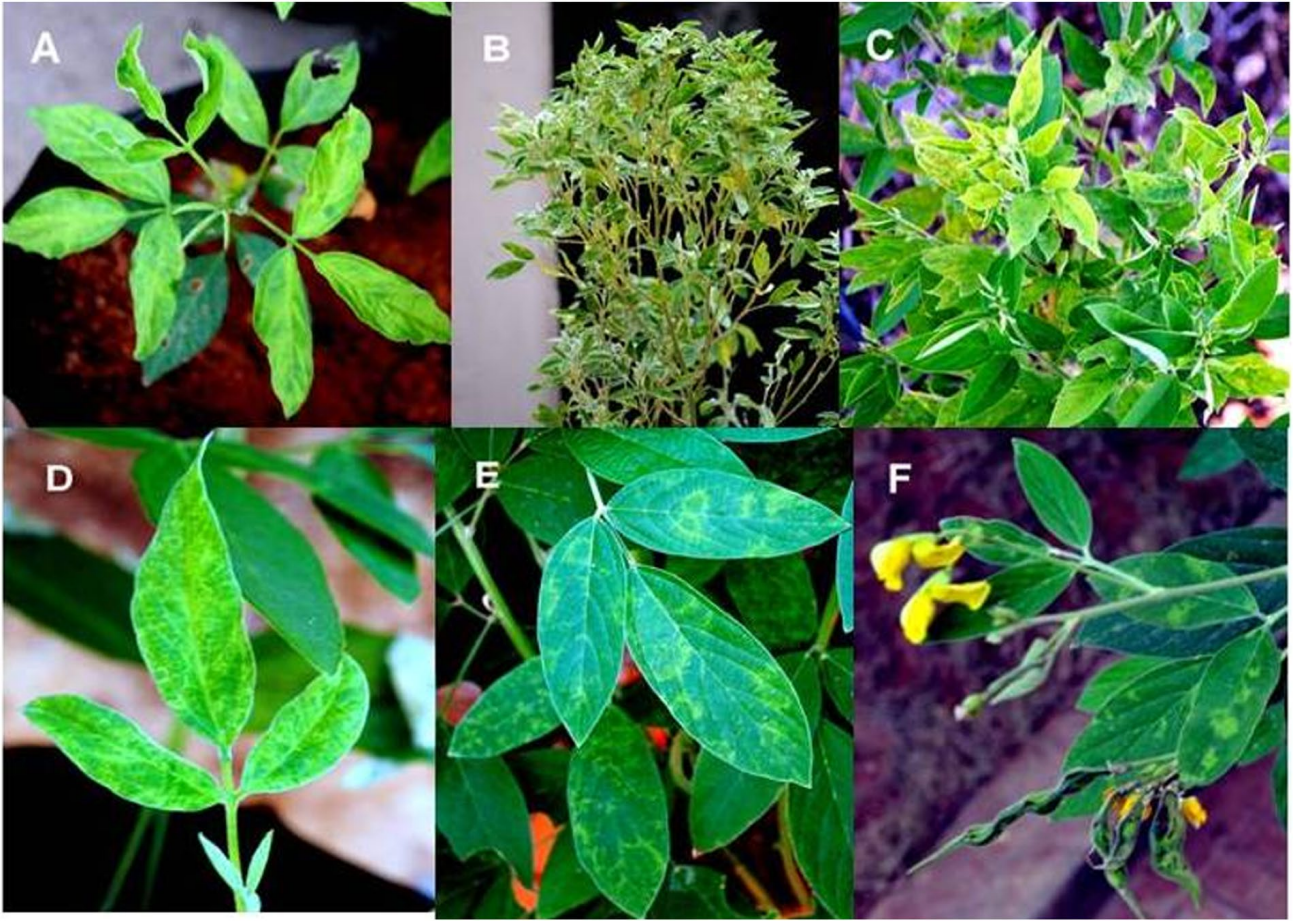
Sterility mosaic virus infected pigeonpea cultivars showing symptom variation: PPSMV-P sub-isolate Chevella inoculated pigeonpea cv. ICP8863 seedling showing chlorosis and mosaic symptoms and typical crinkled emerging trifoliate with mosaic symptoms (**A**) SMD affected pigeonpea cv. *Erra kandulu* in Chevella pigeonpea field, the plant was about 180 days old showing complete sterility and proliferation of vegetative growth (**B**), fresh growth of the ratooned (severe pruning) plant with severe mosaic and chlorosis symptoms in Mg2 pigeonpea field Chevella (**C**), pigeonpea cv. ICP2376 showing ring spot symptoms 15 days after inoculation (**D**), clear ring spot symptoms as the plant was growing with infection (**E**), matured plant with no sterility and foliar ringspot symptoms (**F**).

Pigeonpea sterility mosaic is a major disease of pigeonpea [*Cajanus cajan* (L.) Millspaugh] in the Indian subcontinent. The susceptible genotypes show proliferation of branches, varied degrees of mosaic and mottling on the leaves^1,2^ with a partial or complete sterility of the infected plants (Fig. 1A,B). Whereas pigeonpea genotype ICP2376 develops chlorotic ring spots with no sterility upon PPSMV-P isolate (Fig. 1D–F) infection^3^. The disease is transmitted by its natural vector the eriophyid mite (*Aceria cajani*, Channabasavanna)^4^ in a semi-persistent manner^5,6^. PPSMV Patancheru isolate as well as sub-isolate Chevella (contains PPSMV-I and PPSMV-II) infected ICP2376 cultivar produces ring spot symptoms whereas other isolates previously studied^3^ developed severe mosaic symptoms in the infected plants. This suggests mixed infection of PPSMVs may be the cause of ring spot symptoms coupled with genotype variation in ICP2376, as symptoms caused by individual viruses are not known.

Although this disease was first described 85 years back^7^, efforts made recently gave us a clue about the nature of the pathogen^8–10^, associated with the diseased plants. Purification of sterility mosaic virus as enveloped spherical particles from the infected plants was not successful. However, an association of virus particles was noticed in infected cells identified as double membrane bodies (DMBs)^11^. Similarly, plants infected by different emaraviruses containing the DMBs were not recognized earlier as the disease causing agents. However, *European mountain ash ringspot-associated virus* (EMARaV) and *Wheat mosaic virus* (WMoV) were purified from the infected plants as enveloped spherical particles^12–14^. Purification of sterility mosaic virus from pigeonpea is difficult because of its low titer, besides the SMD infected pigeonpea plants contain interfering polyphenols and secondary metabolites in elevated levels^15^. Obtaining purified intact virus particles facilitate reliable estimates of genome composition especially in a situation where two emaraviruses exist (PPSMV-I and PPSMV-II) in a mixed infection.

Morphologically, all emaraviruses reported till date have virus particles as quasi to spherical with double lipid membrane-bound particles containing four (EMARaV, *Rose rosette virus* RRV and PPSMV-I in this study), six (*Fig mosaic virus* FMV, PPSMV-II in this study) and eight (WMoV, *Raspberry leaf blotch virus* RLBV) segmented negative sense RNAs encapsulated in nucleocapsid and transmitted by eriophyid mites belonging to different genera^14,16,17^. However, *Redbud yellow ringspot virus* ^18^ (RYRSaV), and the newly identified *Actinidia chlorotic ringspot-associated virus* ^19^ (AcCRaV) have five RNA segments. *Woolly burdock yellow vein virus* ^20^ (WBYVV) has not been fully characterized and assumed to be a member of genus *Emaravirus*.

PPSMVs share similarities with other emaraviruses in their genomic organization and conserved 5′ and 3′-end sequences, additional non-conserved RNA segments (in PPSMV-II) which encode nucleocapsid proteins (NCP) to facilitate genome encapsidation, with over all limited sequence homology and eriophyid mite transmission. Plant viruses with these traits have been grouped to an unassigned genus *Emaravirus* ^21^, other members of this genus includes EMARaV the type member of this genus^22^, FMV^23–25^, RRV^26^, RLBV^17,27^, WMoV^14^, RYRSaV^18^, AcCRaV^19^, and WBYVV^20^.

The results of the recent studies of Patancheru isolate revealed the association of PPSMV-1 and PPSMV-2 containing a diverse number of RNA genomic segments, in particular PPSMV-1, which is remarkably differerent from our findings. In this study, we described a workable protocol for analysis of virus-associated dsRNA from pigeonpea host. The analysis of total small RNA populations was performed to characterize the genomic RNA segments and variations in PPSMVs. High-throughput sequencing of small RNA (sRNA) was employed to sequence multiple RNA segments that resulted in establishing the complete genome of PPSMV-I containing four genomic RNAs and PPSMV-II containing six genomic RNAs. Furthermore, their evolutionary relationships were studied with other related viruses by performing phylogenetic analysis. This paper will provide an improved understanding of the molecular biology of PPSMVs, their relationship to each other and with pigeonpea sterility mosaic disease.

## Results

### Eriophyid mite inoculation (PPSMV-P, sub -isolate Chevella)

PPSMV-P sub-isolate Chevella was inoculated routinely and used to obtain 100% infection in the two susceptible cultivars ICP8863 and ICP2376. Eriophyid mite inoculated (Fig. S1) pigeonpea cvs. ICP8863 and ICP2376 produced initial symptoms approximately 10 to 15 dpi. Even in the initial stages symptom variation was noticed between susceptible pigeonpea cultivars (Fig. 1A,D). The inoculated pigeonpea cv. ICP8863 showed typical SMD symptoms with the crinkled emerging trifoliate and visually recognizable typical chlorotic halos and chlorotic ring spot symptoms in pigeonpea cv. ICP2376. PPSMV-I and PPSMV-II were detected from the infected plants of both the cultivars. Interestingly, few- to- many ICP8863 plants showed the presence of only one virus i.e. PPSMV-I and confirmed similar observations made that few SMD affected plants in the field setting also showing segregation of the two viruses^10^. A few times that we got plants with PPSMV-I only did not survive to maturity.

### Survey of SMD affected pigeonpea fields in the three locations

During surveys of Chevella and Bengaluru locations, it was noticed that the cultivars grown in the farmer’s fields were of long duration, medium to tall traditional local pigeonpea varieties, known to be susceptible to SMD and *Fusarium* wilt diseases. Typical SMD symptoms (Fig. 1B) were noticed in the infected pigeonpea plants at the three locations. At TNAU centre, in Coimbatore where susceptible pigeonpea cultivars CO-5, CO-6, and ICP8863 were grown in research farm, the symptoms predictably were uniform, and the disease incidence was about 60%. Pigeonpea cv. *Erra kandulu*, a popular cultivar being cultivated in Chevella area, is known to be susceptible to SMD and *Fusarium* wilt diseases. SMD incidence at this location was about 45% while the disease incidence was 40–45% at Bangalore pigeonpea fields. In off-season survey, 30% of the ratooned plants had aggressive mosaic mottling and chlorosis in MG-2 field of Chevella (Fig. 1C).

### Analysis of dsRNA and total RNA from SMD affected leaves

**Figure 2:**
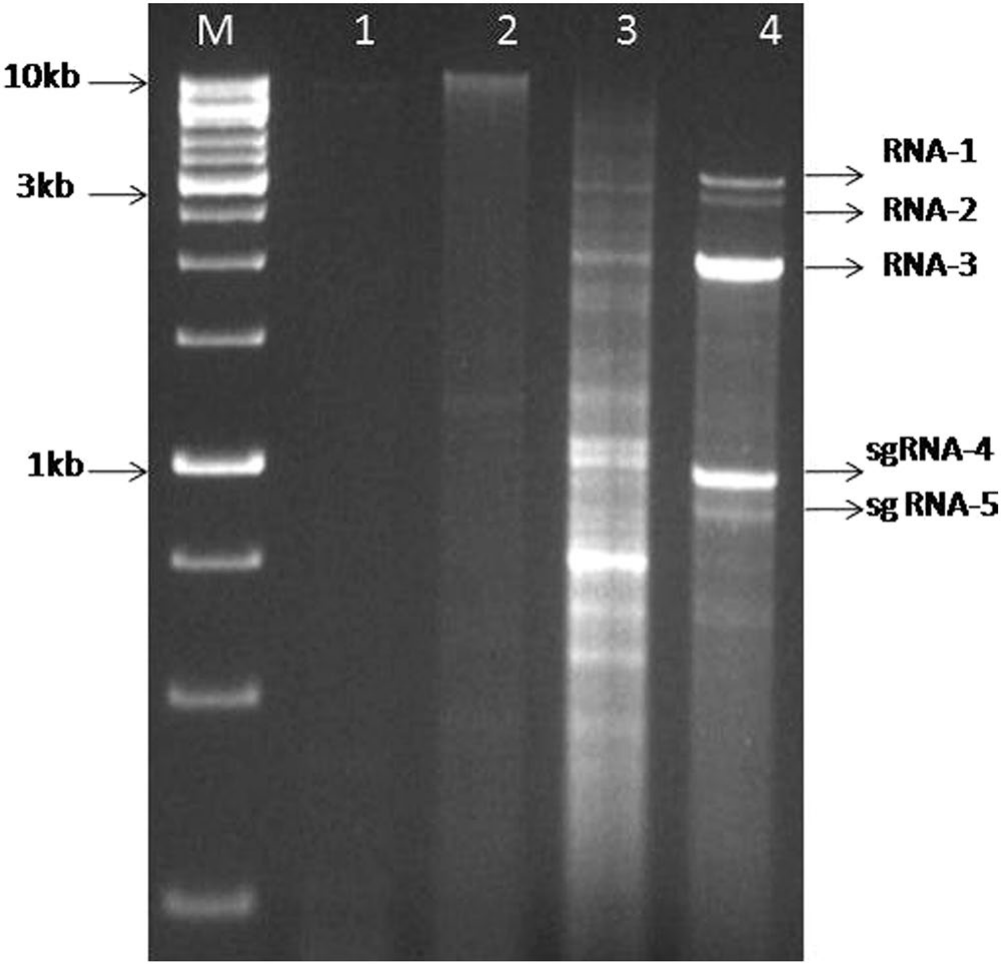
Resolution of dsRNA isolated from SMD affected pigeonpea in 1% agarose gel: dsRNA preparation from non-symptomatic *N*. *tabacum* cv. Xanthi (lane 1) and pigeonpea cv. *Erra kandulu* (lane 2); dsRNA from SMD affected pigeonpea cv. *Erra kandulu* Mg-1 sample, (PPSMV-P sub-isolate Chevella) (lane 3). DsRNA from *N*. *tabacum* cv. Xanthi infected with CMV (Lane 4), used as dsRNA size marker. M- Standard DNA marker.

DsRNA extracted from symptomatic pigeonpea samples infected by different isolates as well as mite inoculated pigeonpea accessions ICP8863 and ICP2376 showed a varied number of dsRNA segments (Figs 2 and S2), while the symptomless pigeonpea leaves did not show similar RNAs. Infected plants with PPSMV-B and PPSMV-C isolates showed six dsRNA segments with sizes ranging from about 7.0, 2.3, 1.8, 1.5, 1.36 and 1.2 kb (Fig. S2), whereas PPSMV-P sub-isolate Chevella infected leaves contain several dsRNA segments including the above mentioned six RNAs and some RNA segments of less than 1 kb (Fig. 2). We tested repeatedly different infected samples from Chevella and noticed consistently more than six dsRNA segments. This indicates that yet another possible mite-transmitted virus could be associated with PPSMV-P sub-isolate Chevella affected pigeonpea cv. *Erra kandulu* field plants. This is confirmed by the analysis of mite inoculated ICP cultivars where a similar number of RNAs was noticed. As mentioned earlier, infected pigeonpea samples from the field may be infected by rare incidence of other viral infections. However, in very few instances of multiple samplings, an association of other dsRNA was noticed while no exclusive RNA of PPSMVs could be detected and hence were discarded (Fig. S2).

### Detection of PPSMV-I using SMV primers

The dsRNA analysis indicated the existence of variation in the viral population (PPSMV-I and PPSMV-II) in the susceptible plants infected by PPSMV-P sub-isolate Chevella. SMV-1 and SMV-2 prime﻿rs^8^ based amplifications employing total RNA from the infected plants resulted in the detection of PPSMV-I from Mg-1, Mg-2, and mite inoculated cv. ICP8863 (infected by PPSMV-P sub-isolate Chevella). The RT-PCR predicted product (321 bp) was amplified in PPSMV-P sub-isolate Chevella infected samples (Fig. S3) but not in the asymptomatic cv. ICP8863. Whereas, SMD affected pigeonpea samples Bng-1, Bng-2, Bng-3 (PPSMV-B isolate) and CO-5, CO-6 and ICP8863 cvs. (PPSMV-C isolate) which was found to contain PPSMV-II alone were negative for 321 bp amplicon, indicating that the SMV oligonucleotide primers are specific, could detect only the PPSMV-I (Supplementary Table 1).

### Distinct genomic components identified

The enriched virus-derived small RNA (vd-sRNA) reads obtained from SMD affected samples of Mg-1, Mg-2, and Bng-1, were assembled to form contigs further used for viral genome assembly. A total of 101 contigs were generated, of which 75 contigs were found specific to viruses by BlastX homology search. For emaraviruses, 62 contigs (23 contigs each of Mg-1 and Mg-2 samples) of PPSMV-P sub-isolate Chevella and 16 contigs from Bng-1 sample of PPSMV-B isolate were generated (Supplementary Table 2). Apart from emaraviruses, hits corresponding to *Tobacco streak virus* (8) and *Bean common mosaic virus* (5) were also obtained in Bng-1 sample. Contigs with a mean length of 404 nucleotides and maximum contig length of 5822 nucleotides were obtained. Homology search revealed that generated contigs showed a relationship with two different members of genus *Emaravirus*. One group of contigs showed homology with RRV and were identified as genomic segments of PPSMV-I and the second group having homology with FMV were recognized as genomic segments of PPSMV-II. Small RNA sequence analysis revealed that the contigs generated from Mg-1 and Mg-2 samples belonged to PPSMV-I and PPSMV-II, while contigs assembled from Bng-1 sample (PPSMV-B isolate) are related to PPSMV-II. Emaraviruses are known to contain conserved complementary nucleotides (13–14 nt) at 5′ and 3′ regions, resulting in the formation of a panhandle structure, and this region was used as a tag for identification of terminals or complete sequence of RNA segment. Based on these results, primers were designed for PPSMV-I and PPSMV-II, and their genomic segments were authenticated by full-length amplification of each RNA segment, cloned and sequenced.

### Genome organization of Pigeonpea sterility mosaic virus-I

**Figure 3:**
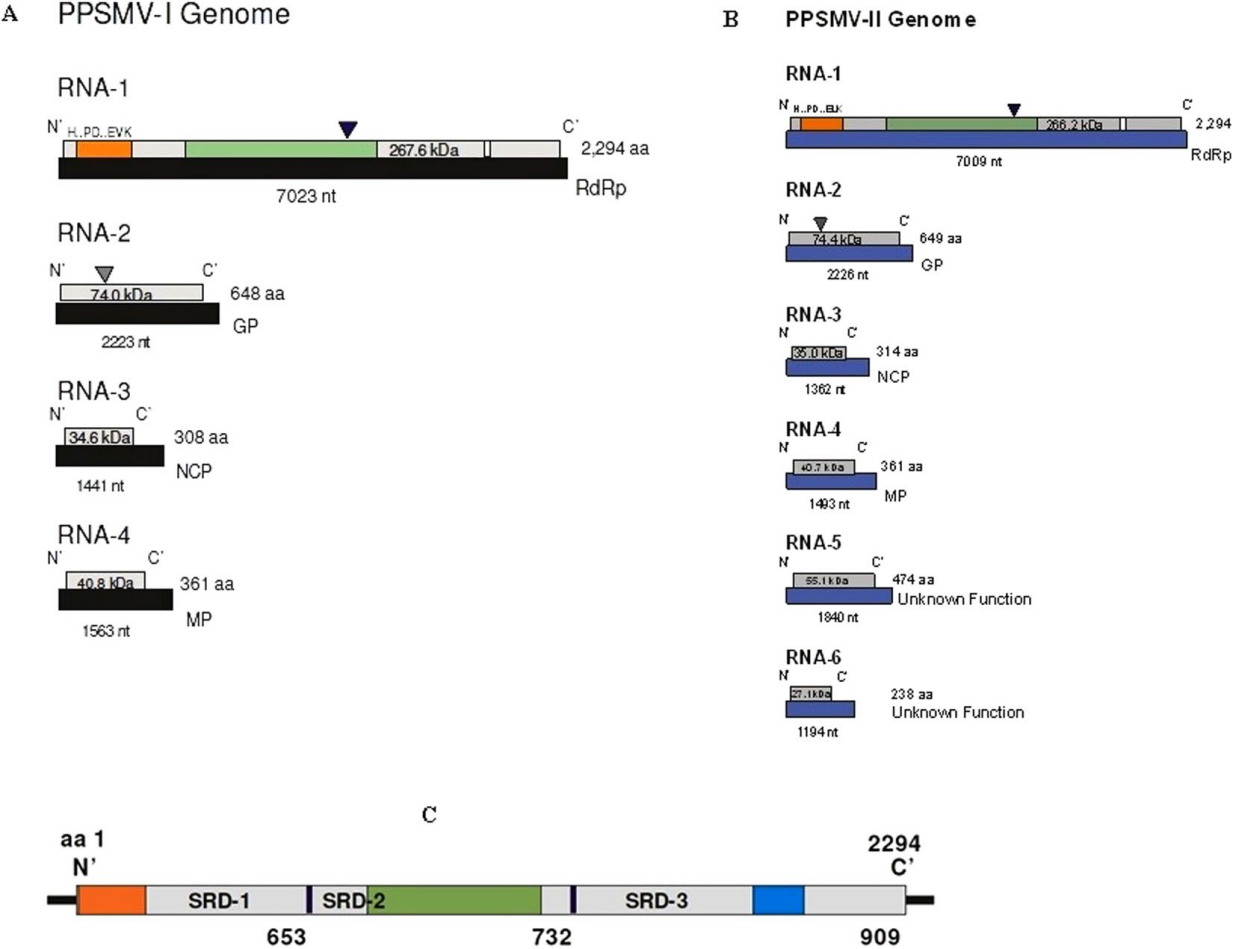
Schematic organization of the genome of pigeonpea sterility mosaic viruses: (**A**) PPSMV-I contains four (-) sense single strand RNA segments and (**B**) PPSMV-II contains six genomic RNA segments. The large RNA-1 encodes RdRp, RNA-2 encoding glycoprotein precursor (GP) and the cleavage site was denoted by gray triangle, RNA-3 encodes nucleocapsid protein and RNA-4 encodes movement protein. PPSMV-II contains additional RNA-5 (55 kDa), RNA-6 (27 kDa) with unknown function. Endonuclease and probable cap binding sites are denoted in orange and white boxes of the RdRp segments of the PPSMVs. The polymerase catalytic motifs A to F were identified in bunya-RdRp central region coloured as green box. Catalytically active aspartate (Asp_1256_) of SDD motif was marked by a black triangle. The RNAs are shown as black and blue boxes and the encoded proteins as grey boxes. (**C**) PPSMV-I and PPSMV-II RdRps are being alike and the line drawing representation is of PPSMV-II which shows imaginary SRD-1, SRD-2 and SRD-3 domains and the location of endonuclease (orange), Bunya RdRp (green) and proposed cap binding site (blue) subunits (See Table 1).

**Figure 4:**
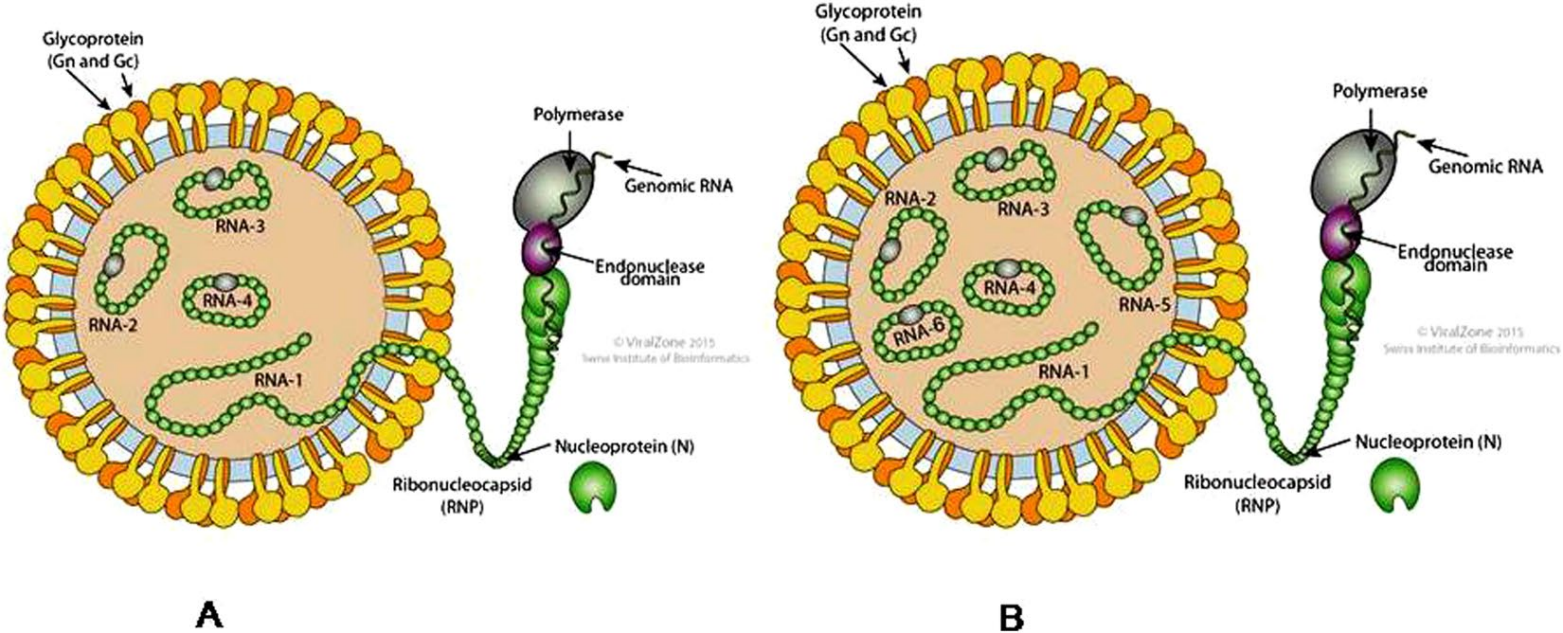
Schematic diagram of particle structure of PPSMVs (ViralZone, Swiss Institute of Bioinformatics), with negative sense ssRNA segmented genome. PPSMV-I (**A**) genome contain four RNAs and PPSMV-II (**B**) genome with six RNA segments.

Following paragraphs describe the nature of the functional four RNAs (1–4) of both the PPSMVs by sequence analysis. The analysis provides evidence, the role they may play in virus multiplication, spreading infection and the variations of RNA-5 among the PPSMV-II isolates. The sequence of each RNA segment was compared with orthologs of known emaraviruses and reasoned that the PPSMVs belong to genus *Emaravirus* while showing the common characters with the members of the larger family *Bunyaviridae*. Sequence analysis showed that PPSMV-I, one of the two viruses identified with PPSMV-P sub-isolate Chevella contains four RNA segments as genome (Figs 3 and 4) like EMARaV and RRV. PPSMV-I RNA segments have key characteristics common to RNAs belonging to emaraviruses including conservation of nucleotides at the terminal ends and with a high probability forming a panhandle structure with complementary ends^16,28–30^. Twelve nucleotides at the 5′-end and the 3′-termini are conserved in all the RNA segments of the genome except RNA-4 where ten nucleotides of the 3′-terminus were conserved. The conservation at 5′-end was almost complete except the least variation, in RNA-2 at nucleotides position, as 5′-A^9^ instead of 5′-G^9^, was noticed. These terminal conserved sequences were used as tag for complete 5′ and 3′ end sequence confirmation of individual RNA segments. As mentioned earlier, the genomic segments of PPSMV-II also have 5′-terminus 13 nts base pairing with the 3′-terminus complimentary ends resulting in a ‘panhandle’ structure with minor variation. Complementary terminal sequences were also observed in other emaraviruses^14,22,31^ and monopartite ss (-) RNA viruses. The signals directing RNA synthesis are localized within the 5′ and 3′ end non-translated terminal regions^32^ and also were attributed to the region responsible for BUNV RNA segment multiplication^31^. The evidence provided thus suggests that these specific sequences in the termini could be involved in the initiation of transcription and also regulating genome replication^31,33,34^.

The negative strand viral polymerases are known to catalyze the transcription of viral mRNAs by a process known as “cap-snatching.” The 5′-cap of cellular pre-mRNA is recognized by the viral RdRp constituent (s) and cleaved 10–13 nucleotides (13–18 in TSWV) downstream of the cap by the endonuclease domain (N-terminal) while interacting with second active cap binding site at the C-terminal end^35–38^ and use them in viral RNA replication. Similar studies were reported in the case of FMV^25^. We have identified conserved residues of cap-snatching and cap binding sites at two different domains of the polymerase of PPSMVs (Fig. 3).

The RNA-1 segment being the largest (7023 nt) (Fig. 3A; Table 1) and encodes for a single open reading frame (ORF) between nt 6,885–51 with 50 and 88 nt long non-translated region (NTR) at 5′ and 3′ ends of a 267.6 kDa protein of 2,294 amino acids and identified as RdRp (P1). The polymerase has 54.2% identity with P1 of RRV followed by 53.9% with PPSMV-II, 53.4% with FMV and 48.5 to 32.5% with the P1 proteins of other members of genus *Emaravirus* (Table 2).

**Table 1:** Genome characteristics of Pigeonpea sterility mosaic viruses.

*Pigeonpea sterility mosaic virus-I*			
*Pigeonpea sterility mosaic virus-II*			
**Genomic segments of PPSMV-I**	**5′NTR**	**Coding region**	**3′NTR**
RNA-1 RNA dependent RNA polymerase (RdRp) 7023nt long, 2294 aa, 267.6 kDa	50 nt	6885 nt-51 nt	88 nt
RNA-2 Glycoprotein 2223nt long, 648 aa, 74.0 kDa	41 nt	1988 nt-42 nt	235nt
RNA-3 Nucleocapsid protein 1441nt long, 308 aa, 34.6 kDa	101 nt	934 nt-102 nt	406nt
RNA-4 Movement protein 1563nt long, 361 aa,40.8 kDa	76 nt	1162 nt-77 nt	401nt
**Genomic segments of PPSMV-II**	**5′NTR**	**Coding region**	**3′NTR**
RNA-1 RNA dependent RNA polymerase (RdRp) 7023nt long, 2294 aa, 267.6 kDa	50 nt	6885 nt-51 nt	88 nt
RNA-2 Glycoprotein 2223nt long, 648 aa, 74.0 kDa	41 nt	1988 nt-42 nt	235nt
RNA-3 Nucleocapsid protein1441nt long, 308 aa, 35.0 kDa	101 nt	934 nt-102 nt	406nt
RNA-4 Movement protein 1493nt long, 361 aa, 40.7 kDa	82nt	1168 nt-83nt	325nt
RNA-5 Unknown protein 1840 nt long, 474 aa,55.1 kDa	109 nt	1534 nt-110nt	306 nt
RNA-6 Unknown protein 1194 nt long, 238 aa, 27.1 kDa	68 nt	785 nt-69 nt	406 nt

**Table 2:** Comparison of percent amino acid identities of genomic proteins pigeonpea sterility mosaic viruses and between the members of genus *Emaravirus*. P1 (RdRp), P2 (GP), P3 (NCP), P4 (MP), P5 and P6 proteins.

*Pigeonpea sterility mosaic virus-I* genome (PPSMV-I Patancheru sub- isolate Chevella)						
Virus	**P-1**	**P-2**	**P-3**	**P-4**	**P-5**	**P-6**
PPSMV-I*	100	100	100	100		
PPSMV-1(P)**	97.04	98.15	99.68	100		
RRV	54.24	43.97	41.50	38.55		
PPSMV-II*	53.92	44.18	41.58	40.78		
PPSMV-2(P)	53.83	43.87	41.58	40.78		
FMV	53.37	45.24	41.58	39.39		
EMARaV	48.33	39.05	35.74	14.52		
RYRSaV	47.98	40	33.44	27.53		
RLBV	33.95	28.72	25.70	20.39		
WMoV	32.55	24.72	22.90	18.29		
AcCRaV	48.47	40.50	35.95	29.58		
***Pigeonpea sterility mosaic virus*** **-II genome (PPSMV-I Patancheru sub- isolate Chevella)**						
EMARaV	49.69	35.76	35.76	19.68	—	—
RYRSaV	46.91	39.37	38.03	32.96	21.53	—
PPSMV-I^#^	53.92	44.18	41.58	40.78	—	—
PPSMV-1(P)	53.65	44.03	41.58	40.78	86.47	—
RRV	68.38	52.26	61.86	60.94	—	—
FMV	73.75	57.72	79.94	74.70	32.77	31.72
PPSMV-II^#^	100	100	100	100	100	100
PPSMV-2(P)	99.61	98.00	98.40	99.45	94.93	97.90
RLBV	34.70	24.84	21.79	17.37	16.16	17.71
WMoV	33.64	23.54	20.37	21.41	19.02	17.01
AcCRaV	47.44	40.09	38.82	31.34	26.53	—

PPSMV-1P** (Patancheru isolate) contains more than four RNA segments and PPSMV-I (Patancheru sub-isolate Chevella) genome was compared with the first four functional RNAs of other emaraviruses. PPSMV-1P** and PPSMV-I* appears to be almost identical. PPSMV-I has closest similarity to RRV followed by PPSMV-II and PPSMV-2P (Patancheru isolate). **In our study, we disputed the claim that PPSMV-1P has five genomic segments. PPSMV-2P and PPSMV-II^#^ (Patancheru sub-isolate Chevella) appears to be almost identical. PPSMV-II^#^ is closely related to FMV in all corresponding RNA segments including RNA-5, followed by RRV, in comparison with other emaraviruses. RLBV and WMoV contain largest genomes with eight RNA segments and we have included the first six of them for the analysis.

**Figure 5:**
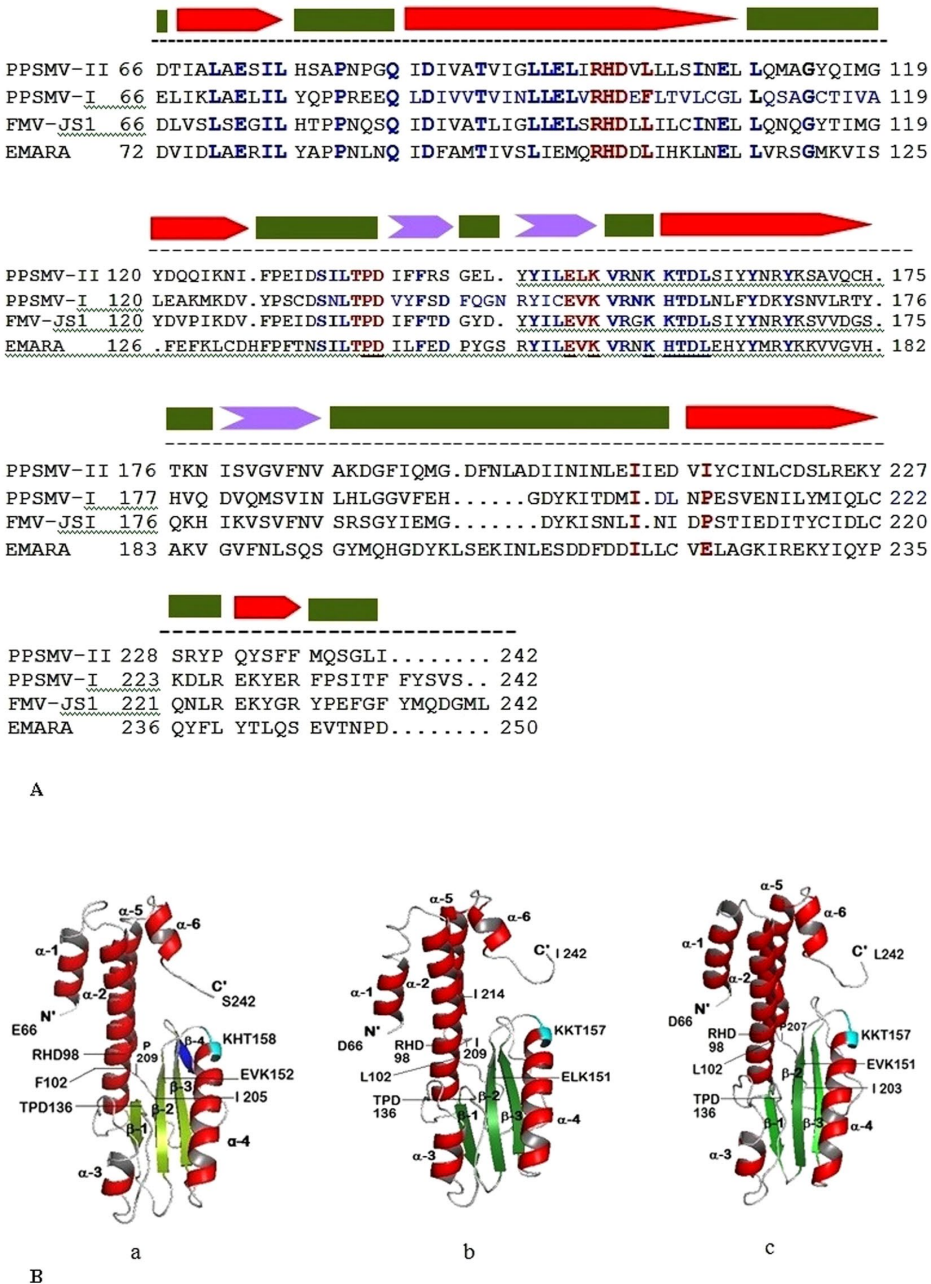
Multiple alignments of amino acid sequence representing endonuclease subunit of members of the genus *Emaravirus* and structural comparisons. (**A**) Structure based sequence alignment of endonuclease subunit of PPSMVs, FMV-JS1 and Emaravirus were aligned amino acid position 66 to 242. Universally and moderately conserved motifs, RHD_98–100_, TPD_136–138_, and ExK_151–153_ (red) known to “cap-snatching” function while almost conserved KxTDL_157–161_ (blue) motif seems exclusive to members of *Emaravirus* genus were identified. Individual active residues and of the conserved motifs are indicated with bold red letters and more than three individual conserved amino acids in a row are coloured blue. (**B**) Comparison of tertiary structures of endonuclease subunit of Emaraviruses. The putative endonuclease subunit located in the SRD-1 domain at the N’-terminal end of RdRp, (orange box) in Fig. 3C. Conserved amino acid sequence of endonuclease subunit of PPSMVs, FMV-JS1 (amino acid position 66 to 242) were aligned with *Bunyamwera virus* (1–185aa) and used to develop the 3D structures of (a) PPSMV-I with 6.7 Å resolution, (b) PPSMV-II with 7.1 Å resolution, and (c) FMV with 6.9 Å resolution. Cartoon representation of the endonuclease of emaraviruses showing overall topology and the architecture, similar to each other and remarkably with *Bunyamwera virus* (1.3 Å resolution) (Fig. S6). The three dimensional structure of endonuclease in general comprised of six α-helices (red) and three β-sheets (green) sustaining the functional motifs. PPSMV-I seems to contain an additional β-sheet 4 (blue) like in *Bunyamwera virus*. Presence of RHD, TPD, ExK and almost similar core, cation-binding fold as found generally in the nuclease super-family were identified in the three emaraviruses. KxTDL motif present in Emaraviruses was identified at the top of α-4 helix (cyan) in the proximity of central core. Starting residues of motifs were numbered.

For convenience, the RdRp is described as containing N-terminus SRD-1, a central region as SRD-2 and C-terminus SRD-3 domains (Fig. 3C) and identified presence of important constituents, endonuclease sequences like *Bunyaviridae* polymerase, and probable cap binding site in these domains in the same order (Table 3). The sequence of conserved residues specific to endonuclease sub-domain known to play a role in “cap-snatching” mechanism was identified. This sub-domain with four motifs (RHD _98–100_, TPD_136–138_ EVK_152–154_ and KTDL_158–161_) present in the SRD-1 domain between 66 to 242 aa (Figs 3A,C and 5) and aligned with *Bunyamwera virus* endonuclease sub domain. While RHD_98–100_ and TPD_136–138_ are universally conserved^37^ containing key cation binding residues and catalytic lysine, variations are observed with regard to other motifs in different genera of *Bunyaviridae* super family. EXK (EVK_152–154_ in PPSMV-I) motif is present in different viruses belonging to *Emaravirus* and *Tospovirus* genera while the later and orthobunyaviruses contain additional DXK motif. KXTDL_158–162_ motif (Fig. 5B-a coloured cyan) remains conserved in the members of genus *Emaravirus* studied (Fig. 5A), and the function of the motif is yet to be determined. Active residues–Ile_105_ and Pro_109_ are present within the endonuclease sub-domain, while Phe_269_ was outside (in PPSMV-I), are known to play a role in endonuclease activity^37,39–43^. These active residues are highly conserved amongst the genera of *Emaravirus*, *Tospovirus*, and *Orthobunyavirus*.

**Table 3:** Salient features identified by bioinformatic analysis.

Protein	Bioinformatic analysis of genomic segments of PPSMVs
RNA-dependent RNA polymerase (RNA-1)	Conserved sequences known to play role in “cap snatching” mechanism was identified at N’-terminal leader sequences in the SRD-1 region of the RdRp. Typical conserved 3D structure of endonuclease has been described A signature divalent cation binding residues occur in core fold of endonucleases in PPSMVs in a pattern H…PD…ExK similar to members of *Orthobunyavirus* and *Tospovirus* genera
	3D pol structure has been studied in the SRD-2 central region of RdRp with characteristic Fingers, Palm and Thumb subdomains and functional conserved motifs A-F in both PPSMVs were identified
	Moderately conserved (between PPSMVs) stretch of thirteen amino acid sequence residing at the same positions in the SRD-3 domain at the C’-termini of RdRp that are similar to cap binding described for other Bunyaviruses were identified. Other emaraviruses; FMV, RRV and EMARaV also showed these sequences
Glycoprotein (RNA-2)	An in depth study of the 3D structure for glycoprotein precursor of both the viruses has been conducted. We showed the croissant shaped precursor protein with a panhandle containing several attributes including the putative cleavage sites and “Phlebovirus” motif
Nucleocapsid protein (RNA-3)	Conserved residues in RNA-3 of the PPSMVs and probable RNA binding region were identified
Movement protein (RNA-4)	The RNA-4 of both the PPSMVs encodes respective P4 proteins assumed involved in cell-to-cell movement. Highest sequence similarity of PPSMV-II with FMV homolog was noticed. The P4s contain different elements in sequential order; the N’-terminal signal peptide, the TMV 30 kDa movement protein domain which showed typical structural pattern of α-helices and β-sheets in its secondary structure. Presence of likely DnaK domain in the C-terminal half have been identified. The conservation (I/V and D) at the N-terminal end showing beta-1 and beta-2 sheets seems almost universal in members of 30 K family of MPs, is critical for the movement function

The signature divalent cation binding residues occur in core fold of endonucleases in a pattern as described for *Orthobunya-* and *Tospovirus* genera (H….D…PD….DxK…..T), the pattern with the PPSMVs, FMV-JS1, and Emaravirus appear to be H…PD…ExK as of now till the confirmations are made (Table 3, Fig. S5A). FMV endonuclease also showed conserved sequence sites referred above, and the virus generates 5′ capped mRNAs via cap-snatching, similar to strategies used by other negative-sense multipartite ssRNA viruses^24,25^. Cap snatching is employed by representative viruses of all five genera of *Bunyaviridae* super family^36,37,44^. Taken together, different aspects of our findings point to the fact that PPSMVs like FMV could employ cap-snatching, indicating that this mechanism could be used by polymerase in the RNA synthesis of viruses belonging to genus *Emaravirus*.

Conserved residues identified in the central SRD-2_653–1384_ domain of the RdRp of PPSMV-I resemble the sequences of *Bunyaviridae* polymerase (Fig. 3A,C as a green box in RNA-1). Six highly conserved amino acid sequence motifs (A to F) with active residues exclusive to polymerases were identified in the central region (Fig. S4) and are also present in other members of the *Emaravirus* genus; motif A (SSDASKWSARD_1127–1137_), motif B (NWLQGNLNMISSFVH_1211–1225_), motif C (SDD_1255–1257_), motif D (ITLNEKKTY_1298–1306_), E (EFL_1313–1315_) and F (KDQRTAXDREIYTGNAQ_1049–1065_) (Fig. S5B). These active sites representing the replicative complex are known to play as catalysts by rNTP selection, binding, replication-elongation in series of activities in viral replication^22,45^. These conserved sites of the polymerase are shown in a multiple alignments of amino acid sequence of PPSMVs compared to the members of different genera of *Bunyaviridae* super family (Fig. S5).

A stretch of thirteen amino acid with sequence DPLTYNYWVMPTN_1947–1959_ residing in the SRD-3 domain at the C-terminus of PPSMV-I polymerase was identified for the first time as a probable cap binding site (CBS) (Fig. 3C). The CBS present in this subdomain is not generally conserved across the members of super family *Bunyaviridae* but does so within closely related strains^35^. However, the CBS shared the presence of aromatic amino acids, in particular tyrosine and tryptophan, known to interact with m^7^G of the cap in other cap binding proteins^35,46^. The thirteen-residue motif of PPSMV-I SRD-3 domain showed aromatic residues Y_1951, 1953_ and W_1954_ described essentially for cap binding in vaccinia and influenza viruses. PPSMV-II polymerase also contains similar sequence DNRVYNHWLIPTN_1948 to1960_, having similar cap binding residues Y_1952_ and W_1955_. The CBS is modestly conserved between the two sterility mosaic viruses. However, the predicted CBS of PPSMV-II is identical with FMV (DNRVYNHWLIPTN_1951 to 1963_) and RRV (DHRVYNHWLIPTN_1930 to 1942_) except that RRV is containing the second residue as H_1931_^24,26^. Conserved sites of the endonuclease and probable cap binding sub domains were identified in SRD-1 and SRD-3 domains of PPSMV-I polymerase, primarily by the sequence alignment with members of the *Bunyaviridae* super family.

### 3D structure of endonuclease

The tertiary structure of the endonuclease was developed to examine the topology resemblances and the distribution of highly conserved motifs and individual conserved residues mentioned earlier, between PPSMVs, FMV and *Bunyamwera virus*. The N-terminal sequence with amino acid position 66 to 242 (185 aa) was used to develop the 3D structures of the endonuclease. As shown in Fig. 5B, the structures were resolved at 6.7 Å for PPSMV-I, 7.1 Å for PPSMV-II and 6.9 Å for FMV employing the Zhang lab server^47^. Cartoon representation of the endonuclease model of the above viruses showing overall topology and architecture of the protein folding resembles the endonuclease of *Bunyamwera virus* ^48^ resolved at 1.3 Å (Fig. S6), comprising of six α-helices (red) and the three β-sheets (green). PPSMV-I has additional β-sheet 4 (blue) (Fig. 5B-a). The conserved motifs and key individual residues of PPSMVs (see SRD-1 section) were noticed in the three-dimensional structures of endonuclease. Briefly, presence of the RHD, TPD, and ExK (H…PD…ExK) motifs and almost similar core cation-binding fold as found in the nuclease super-family (H…PD…D/ExK) was noticed in PPSMVs and FMV (Figs 5B a–c; SI 5A). The β-sheet 2 containing the conserved ExK motif present at the central core is surrounded by active residues Leu_102_, Ile_105_, and Pro_109_. Also, KxTDL motif which is conserved in emaraviruses has been located at the top of the α-helix 4 (labeled as KxT), not far away from the center. The endonuclease of PPSMVs, beside their close structural similarity with *Bunyamwera virus*, also showed similarity with analogs found in the PDB library; *La Crosse Orthobunyavirus* (LACV; 5AMQ, 2XI5), *Influenza virus* A (2W69), B (4WRT) and C (5D98), *Pichinde virus* (4I1T) and *Lassa virus* (4MIW) of genus *Arenavirus*.

**Figure 6:**
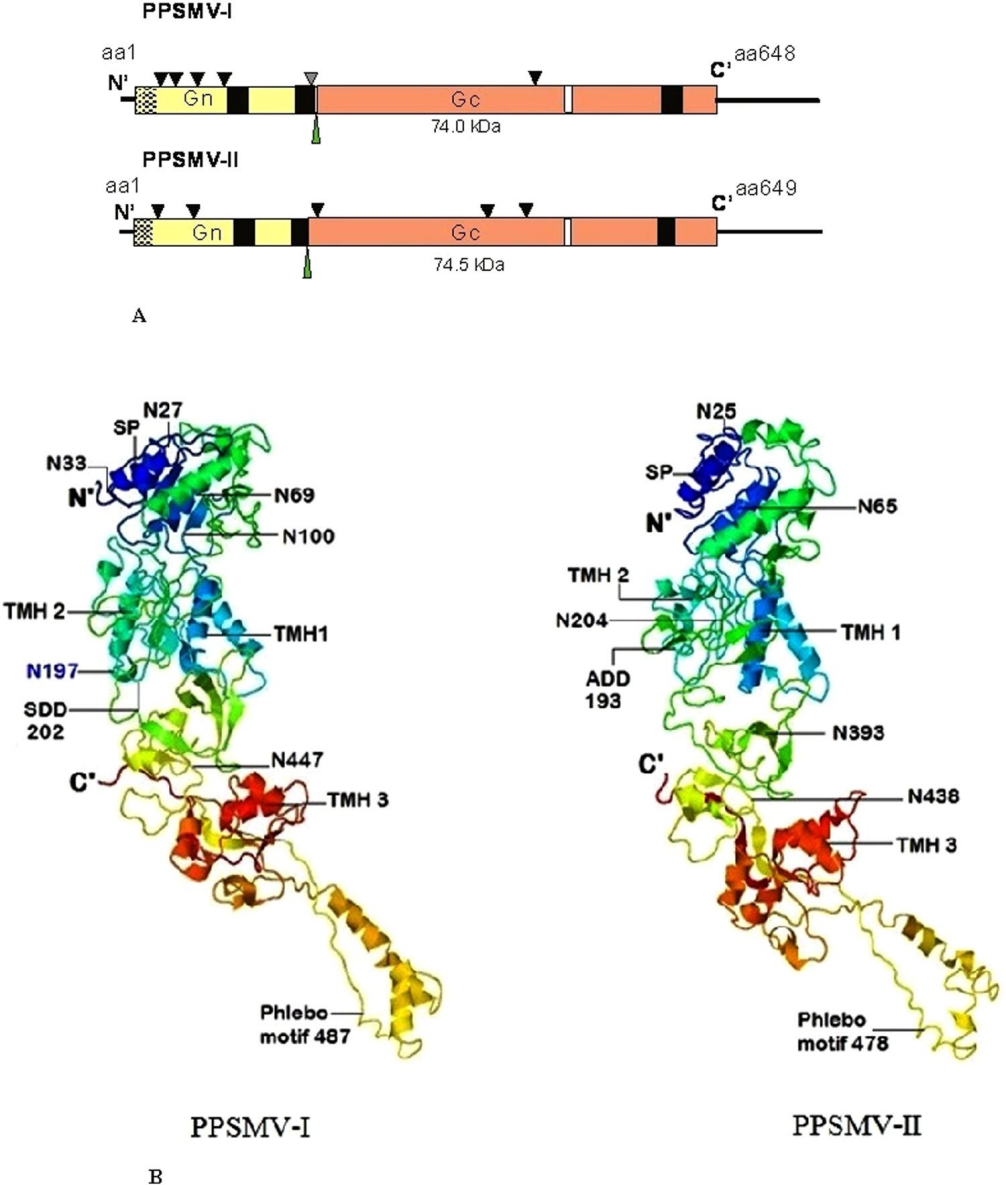
Schematic representation of RNA-2 and structural organization encoded Glycoprotein precursor of sterility mosaic viruses: (**A**) RNA-2 of PPSMV-I and II encode a 74 kDa and 74.5 kDa glycoprotein precursor (P2), respectively. Gn and Gc peptides of the precursor are represented by yellow and orange boxes and the cleavage site was shown by a green narrow triangle. The black line represents the NTRs. The signal peptide sequences are indicated (in mesh). Black boxes represent the transmembrane helices (TMH). Potential glycosylation sites are marked as black triangle along with the NPS tripeptide as gray triangle PPSMV-II P2. The Phlebovirus glycoprotein like motif is indicated in white boxes. PPSMV-I contains maximum number of glycosylation sites in Gc peptide portion. (**B**) Structural organization of glycoprotein precursor: Cartoon representation of glycoprotein precursor of PPSMV-I molecule with 12.1 Å, resolution (left) and PPSMV-II, 12.7 Å, (right). 3D structure of precursor protein contains 18α- helices and 17 β-sheets each is shaped like a croissant with panhandle. Position of signal peptide (SP, deep blue α-1 helix), glycoprotein precursor Gn, Gc cleavage sites (SDD202, AKD193), glycosilation (N) sites and location of transmembrane helices (TMH) were labled. The phlebo virus like motif in both the viruses is identified in the panhandle of the protein. FMV and RRV glycoprotein precursors also have identical characterstic archtecture.

RNA-2 is 2,223 nt long and encodes a single ORF between nt. 1988 to 42 with 41 and 235 nt NTR at 5′ and 3′ ends of a 74 kDa (P2) protein of 648 amino acids (Fig. 3A, Table 1). RNA-2 encoding protein was identified as a glycoprotein precursor (GP). By the computer assisted analysis, sequences specific to glycoprotein functioning have been identified. These functional moieties noticed in the PPSMVs resembled the ones present in the viruses belong to the family *Bunyaviridae*. The N-terminal region of the glycoprotein precursor of PPSMV-I contains a signal peptide cleavage site, located at amino acid position 22 and 23 (AMA↓S) (SignalP- 4.1 Prediction (http://www.cbs.dtu.dk/cgi-bin, EUK Networks)^49^. The glycoprotein precursor yields a dimer having a putative cleavage site at FS↓DD, (Ser_201_ and Asp_202_), and the precursor may be processed into two Gn (22.4 kDa) and Gc (51.6 kDa) smaller glycoproteins. All emaraviruses have been shown to have the processing site except in RLBV glycoprotein, in which case the strategy of cleavage process remains to be identified^27^. The glycoprotein contain three transmembrane helices (TMH) at amino acid positions 103–125, 178–200 and 593–615 (TMHMM v.2.0 server^50^) (http://www.cbs.dtu.dk/cgi-bin). Six potential glycosylation sites were identified at amino acid positions 27, 33, 69, 100, 197 and 447^51^. The analysis predicted a potential fifth glycosylation site close to the putative processing site amino acid position 197 having a sequence NPS (represented by a grey triangle; Fig. 6A). PPSMV-I glycoprotein precursor seems to be exceptional by having five out of six glycosylation sites being located in the Gn peptide (Fig. 6A), a similar organization was not observed in glycoprotein of other emaraviruses. Most viruses have more glycosylation sites in the Gc glycoprotein including PPSMV-II. The analysis revealed a conserved motif that was identified in glycoprotein precursor of phleboviruses of the family *Bunyaviridae* is present in the glycoprotein precursor of PPSMV-I. This motif GCYDCQNG_487–494_ is not well conserved amongst the emaraviruses. PPSMV-I glycoprotein precursor has 44.2% amino acid similarity with PPSMV-II, 45.2% FMV P2 and percent similarity of 44 to 24.7 with the P2 proteins of other emaraviruses, the lowest similarity was observed with WMoV (Table 2).

PPSMV (now PPSMV-1) Patancheru isolate P2 sequence published from the previous investigation was partial with 623 amino acids (HF568802) making it thus shorter by missing N-terminal 25 amino acids which contain signal peptide cleavage site. However, the characteristics of precursor described, reflects of a full-length glycoprotein precursor^9^. Description of peptide cleavage or signal peptide site information of PPSMV-1 was different from our findings, and the authors reported four out of six glycosylation sites. Furthermore, there was the substitution of 10 amino acids. This precursor protein has an amino acid similarity of 98.15% with PPSMV-I P sub-isolate Chevella (present study).

### The 3D structure of glycoprotein precursor

PPSMV-I glycoprotein precursor and its three-dimensional structure was studied employing the Zhang lab server^47^. The 3D structure was solved at the 12.1 Å resolution and the structures developed in sufficient quality to conclude that various structural elements are similarly arranged to those of PPSMV-II, with an average resolution of 12.7 Å. The precursor proteins of both the viruses showed similar topology with no significant differences in the architecture. PPSMV-I glycoprotein precursor contains 21 α-helices and 17 β-sheets and is shaped like a croissant with panhandle where the croissant and panhandle correspond to the Gn and Gc domains, respectively. There is a clear boundary between the two domains. The N-terminal domain (croissant) is packed compact containing the two transmembrane helices (TMH-1 and TMH-2), the specific glycosylation sites and the panhandle containing the third TMH and the conserved eight residues Phlebo-like motif close to the α-helix 13 (in PPSMV-II, α-helix 14) (Table 3). The C-terminal folds back towards the peptide cleavage loop (Fig. 6B). PPSMV-II also showed similar feature with 24 α-helices and 16 β-sheets.

RNA-3 is 1,441 nt. long and encodes a single ORF between nt 934 and 102 with 101- and 406-nt NTRs at the 5′ and 3′ends, of a 34.6 kDa (P3) protein of 308 amino acids (Fig. 3A, Table 1). RNA-3 encodes a putative nucleocapsid protein (NCP) and share a group of conserved amino acid sequence motifs, NVLSFNK_134–140_, NRLA_183–186_ and GYEF_204–207_ with the NCP of PPSMV-II and members of the genus *Emaravirus* ^14,16,22–24,26^ predicted to be involved in RNA-binding^27^ (Table 3). In general, these motifs are modestly conserved, rearrangement and substitutions of “active residues” (GYEF) were noticed in the heterogeneous P3-A and P3-B nucleocapsid proteins of WMoV^14^. The nucleocapsid protein of PPSMV-I has 41.6% amino acid homology with PPSMV-II P3, 41.6% with FMV and 41.5% to 22.9% with the P3 proteins of other Emaraviruses (Table 2). Several amino acid residues were identified **(** http://www.imtech.res.in/raghava/pprint/), distributed in N- and C-terminus, predictably play a role in RNA binding^52^.

The RNA-4 of both the PPSMVs encodes respective P4 proteins assumed to be involved in the cell-to-cell movement, a crucial step in plant virus infections. To support this hypothesis, conclusive evidence was provided from the study of related P4 proteins; FMV and RLBV which have been implicated in cell-to-cell movement^27,53,54^. In the present study, the P4 sequences of PPSMVs were analyzed and aligned with other emaraviruses, revealing characteristics that are common to emaraviruses. The P4s contain different elements in sequential order; the N-terminal signal peptide (SignalP 4.0^49^), the TMV 30 kDa movement protein domain similar in its sequence and secondary structure. Presence of likely DnaK domain in the C-terminal half and sequences that have been predicted to be RNA binding elements **(** http://www.imtech.res.in/raghava/pprint/) were identified.

RNA-4 is 1,563 nt long, with a single ORF (nt 1162 to 77) of a 40.8 kDa protein (P4) of 361 amino acids. RNA-4 contains 76 nt and 401 nt as the 5′ and 3′ (unusually long) NTRs, respectively (Fig. 3A, Table 1). The signal peptide cleavage site was detected at amino acid position 18 and 19 (TSG↓D) (Table 3). The multiple sequence alignment of the P4 with the six orthologs of the emaraviruses (PPSMV-II, FMV JS-1, RRV, RYRSaV, RLBV, AcCRaV and WMoV) revealed detectable sequence homology between PPSMV-I P4 with those analyzed (Fig. S7). The central region of the P4 is conserved (I-70 to T-199), identified as TMV 30 K MP^53,55–57^ starting with LP-I_68–70_ to SWKT_195–199_. The 30 K domain containing sequences coding for the highly conserved motifs aligned in all emaraviruses (Fig. S7) including about 40 amino acid (TAV segment) residues. The sequences of RLBV and WMoV are less conserved in comparison with the six other emaraviruses. P4 sequence alignment of eight emaraviruses revealed considerable conservation in the 30 K domain and consensus residues in few motifs PDE_201–203_, QWDVFTFPR_211–219_, SGK_229–231_, QLED_254–257_, GLT/NK_269–172_ detected in the so-called DnaK molecular chaperone region at C-terminus are modestly conserved in majority of emaraviruses and again with low similarity shown by RLBV and WMoV (more details in PPSMV-II P4 section) (Table 3). The P4 peptide sequence of PPSMV-I showed 100% similarity with PPSMV (Patancheru isolate: HF568804). The P4 possesses 40.8% amino acid similarity with PPSMV-II P4, 40.0% of FMV and percent similarity of 38.6 to 14.5% with the P4 proteins of other emaraviruses (Table 2).

### Genome organization of Pigeonpea sterility mosaic virus-II

Six negative-sense single strand genomic RNAs were identified forming the genome of PPSMV-II (Figs 3B and 4B), with a genome size of 15.13 kb which is larger than PPSMV-I (12.25 kb). A stretch of thirteen nucleotides conservation in PPSMV-II RNA segments, at the 5′- and 3′-termini was complete with the exception of RNA-5 where the two adenines 5′- A_8_-A_9_ which are conserved in rest of five RNAs were replaced by T_8_-T_9_. Furthermore, the 5′- and 3′-termini complementation was found to be complete to form the putative panhandle with the exception of RNA-5 where the complementation was interrupted by TT replacement at the 5′-end. The four functional RNA segments of both PPSMVs share several common characteristics, while PPSMV-II showed a close relationship to FMV^23,24^ as they both uniquely contain six RNA segments, confirming the recent observation^10^.

The RdRp (RNA-1) of PPSMV-II is 7009 nt long and slightly smaller to RNA-1 of PPSMV-I (7022 nt) and encodes for a single open reading frame (ORF1) between nucleotides 6930–46 with 45 and 79 nt long NTRs at 5′ and 3′ ends (Table 1). ORF1 encodes the 266.22 kDa protein (P1) of 2294 amino acids (Figs 3B and 4B). The organization of domains (Fig. 3C) (SRD-1 to SRD-3) of the RdRp was same as described for PPSMV-I and were aligned to that of PPSMV-I with predicted functionally equivalent sub units. As detailed in the PPSMV-I RdRp, the conserved sites of cap snatching subunit of the N-terminus and the proposed conserved 13 amino acid residues (DNRVYNHWLIPTN_1948–1960_) as cap binding site could be identified in SRD-1 and SRD-3 domains of polymerase, respectively. The “cap binding” residues are fully conserved with the FMV while moderately conserved with PPSMV-I. Conserved residues of four motifs (RHD_98–100_, TPD_136–138_ EVK_152–154_ and KTDL_158–161_) in the SRD-1 domain representing the putative endonuclease, between nucleotide position 66 to 242 (Fig. 5A) were structurally aligned with FMV, PPSMV-I and *Bunyamwera virus*. The EXK (ELK_152–154_ in PPSMV-II) motif was also identified along with KXTDL_157–161_ motif (Fig. 5B-b, coloured cyan) and the individual conserved residues observed in PPSMV-I. The three dimensional model of endonuclease of PPSMV-II with 7.1 Å resolution was found identical with the endonuclease of FMV and PPSMV-I (Fig. 5B-b,c) and the details mentioned in narration of PPSMV-I endonuclease 3D structure of endonuclease section.

The SRD-2 domain (Fig. 3C) containing Bunya polymerase sequence includes crucial motifs in the central region, similar to polymerase of PPSMV-I described. The conserved residues of motif A (SSDASKWSARD_1127–1137_), B (SNWLQGNLNMISSFVH _1210–1225_), C (SDD_1255–1257_), D (ITLNEKKTY_1298–1306_), E (EFL_1313–1315_) and F (KDQRTAXDREIYTGNAQ_1049–1065_) were identified (Table 3). These active sites representing the replicative complex are known to play as catalysts by rNTP selection, binding, replication-elongation in series activities in viral replication^16,65^ as described in the previous section. The 3D pol structure PPSMV-II revealed (residues 652 to 1400), as closed right hand resembling the 3D pol of typical picornaviruses. The 3D pol contains the conventional sub-domains as fingers, palm, and thumb containing the above, referred as A to F sequence motifs (Fig. S4). The 3D pol of PPSMV-I also has an identical structure (Data not shown). Similar to PPSMV-I RdRp, a probable “cap binding” site with residues DNRVYNHWLIPTN_1948 to 1960_ in the SRD-3 domain of C-terminus of the polymerase was identified. The PPSMV-II polymerase has amino of 53.92% amino acid identity with PPSMV-I P1, 73.75% with FMV and percent similarity of 68.38 to 33.64 with the P1 proteins of other emaraviruses (Table 2).

RNA-2 is 2296 nucleotide long and contains a single ORF between nt 1997–47 nt with 47 and 229 nt long 5′ and 3′ NTRs respectively. This ORF encodes a 74.41 kDa protein (P2) of 649 amino acids (Fig. 3B, Table 1). The P2 has been identified as glycoprotein precursor and yields a dimer having a putative cleavage site at KA↓DD, (Ala_192_ and Asp_193_), at which the precursor may be cleaved into two single glycoproteins Gn (22.1 kDa) and Gc (52.3 kDa). In the N-terminus region, a potential signal peptide cleavage site between Gly_19_ and Thr_20_ was detected. Additionally, the GP precursor contains three transmembrane helices^50^ (TMH) at amino acid positions 109–130, 175–192, and 583–599. Five potential N-glycosylation sites at amino acid positions 25, 65, 204, 393 and 438 were predicted, unlike in PPSMV-I, these sites were evenly distributed (Fig. 6A). Like in PPSMV-I and other emaraviruses, a Phlebovirus glycoprotein containing motif GCYDCQSG_478–485_ is identified in the Gc peptide (Table 3). The PPSMV-II glycoprotein has 44.2% amino acid identity with PPSMV-I, 57.7% FMV P2 and percent similarity of 52.3 to 23.5 with the P2 proteins of other emaraviruses, the lowest similarity was observed with WMoV (Table 2).

RNA-3 is 1362 nt long and encodes a 35 kDa nucleocapsid protein (NCP) of 314 amino acids. RNA-3 encodes a single ORF (P3) between nucleotides 1046 to 102 and contains a 101 and 316 nt NTRs at 5′ and 3′, respectively (Fig. 3B, Table 1). PPSMV-II P3 peptide is longer by six amino acids than PPSMV-I. The putative NCP of PPSMV-II showed a high degree of sequence similarity with the nucleocapsid proteins of FMV and RRV while sharing a conserved long stretch of amino acid sequence. Like the PPSMV-I the putative NCP contain identical conserved amino acid sequence motifs, NVVSFNK_130–136_, NRLA_178–181_ and GYEF_199–202_ (Table 3). The P3 polypeptide protomer has a definite structural folding with long coiled coil N-terminal end (40 residues) on one side of the molecule and the C-terminal tail on opposite side with a central cleft flanked by the N- and C-terminus lobes. This organization makes it possible, protomer to interact with neighboring protomers to form a tetrameric to hexameric ring^58–60^ facilitating the development of viral NCP-RNA complex (encapsidation). The three motifs were identified around the cleft of the protein. The α-4 helix containing NVVSFNK motif, GYEF motif with α-7 helix and NRLA in a coil between α-5 and α-6 helices (data not shown). The bindN analysis predicted stretches of amino acid sequences at N- and C-terminal regions as potential RNA binding sites (Table 3). The nucleocapsid protein of PPSMV-II has 41.6% amino acid similarity with PPSMV-I, 80.0% FMV P3 and percent similarity of 62.0 to 20.4 with the P3 proteins of other emaraviruses (Table 2).

RNA-4 is 1493 nt. long with a single ORF (1168 to 83 nt) of a 40.7 kDa protein (P4) of 361 amino acids. RNA-4 contains 82 and 325 nt at the 5′ and 3′ NTRs, respectively (Fig. 3B, Table 1). The signal peptide cleavage site was detected at amino acid position 19 and 20 (TSG↓M). RNA-4 encodes a putative movement protein (MP). Multiple sequence alignment of the P4 with the other emaraviruses mentioned earlier resulted in almost similar pattern described in PPSMV-I, RNA-4 section including few conserved residues and motifs aligned on both the ends of highly conserved central 30 K domain (Table 3). The conserved sequence of the central region (LPI-_67–69_, ASVA-_90–93_, WTPT-_96–99_, DxR-_113–115_ and SWKT-_196–199_) including at least, parts of TAV segment (T_144_ to M_183_) of this domain is mostly homologous to all the emaraviruses except for RLBV and WMoV (Fig. S7). The TAV segment of PPSMV-II is more homologous to FMV and RRV. The P4 of PPSMV-II, FMV, and RRV share conserved amino acid sequence motifs spanning the entire length of the protein, in comparison with RLBV and WMoV, which shared sequences among themselves.

The 131-residue region (LPI_69_-SWKT_199_) was identified (Fig. S7) to contain sequences and its secondary structure similar to the MPs of 30 K superfamily^53,57^. Its N-terminal end showing β-1 and β-2 sheets containing conserved hydrophobic sequences of aliphatic amino acids followed by a structurally aligned sequence of hydrophobic residues highlighted (Fig. S8) by highly conserved aspartate residue (D-113 in PPSMV-II) at the C-terminus of β-sheet 2, is critical for the movement function^53^. The conservation (I/V and D) seems almost universal in members of the 30 K family of MPs, including seven emaraviruses. The conserved aliphatic residue I106 of the conserved β-1 sheet region (Ile and Val in PPSMVs) has been shown in RLBV to mediate membrane association^53^. It was demonstrated that the conserved residue D127, (equivalent D114, D113 in PPSMVs) is critical for both the movement activity and localization to plasmodesmata of RLBV P4. Examination of the secondary structure of 30 K region of PPSMVs P4 revealed a similar structural pattern of α-helices and β-sheets connected by the coils as predicted earlier^53,57^. We used the same nomenclature to describe the secondary structure, from N- to C-terminus. The PPSMVs P4 structural patterns are almost identical with RLBV and FMV. RLBV P4 protein has a secondary structure identified in the 30 K domain as a *α*A, β*-*1, β-2, *α*B, β*-*3, β-4, β*-*5, β-6, and β*-*7^53^. However, minor differences from the mentioned pattern do exist in PPSMVs. The absence of α-helix ‘*α*B′ between β-sheet 2 and 3 in PPSMV-I and presence of one additional β-sheet-8 in PPSMV-II was noticed.

P4 protein of both the PPSMVs consisting of several single and pairs of amino acids including non-conserved seven (PPSMV-I) to eight (PPSMV-II) amino acid motifs were detected as RNA binding elements^52^. The majority of predicted RNA binding motifs are presented in the C-terminus half of P4 of both the viruses (Table 3). Three motifs MSVKQR_81–86_, RNPTE_116–20_ and GKKRWDKNA_121–129_ belonging to PPSMV-I and single motif NDGIK_115–119_ of PPSMV-II were modestly conserved in the 30 K domains. The P4 peptide sequence of PPSMV-II showed 99.5% amino acid identity with PPSMV (Patancheru isolate: HF912246) and 40.8% similarity with PPSMV-I P4. The P4 possesses 74.7% with FMV and percent similarity of 61.0 to 17.4 with the P4 of other emaraviruses (Table 2). While the lowest sequence similarity was with WMoV, EMARaV, and RLBV. Non-conservation of distinct motifs or lack of them in RLBV and WMoV suggest that the MPs of these viruses belong to two diverse groups (Fig. S9). Taken together, the composition of conserved amino acid motifs and similarity in the arrangement of sequence domains and the similarity in secondary structure convincingly suggest that MPs of PPSMVs also are the members of the 30 K superfamily.

RNA-5 is 1840 nt long, and contains a single ORF between nt 1534 and 110 with 109 and 306 nt long 5′ and 3′ NTRs, respectively (Fig. 3B, Table 1). This ORF encodes a 55.1 kDa protein (P5) of 474 amino acids. The P5 peptide sequence neither share similarity with known proteins nor 5′ and 3′-terminal NTRs with rest of the genomic segments and any known functional domains.

RNA-6 is 1194 nt long, encodes a 27.1 kDa protein (P6) of 238 amino acids with an ORF between nt 785 and 69 (Fig. 3B, Table 1). PPSMV-II RNA-6 contains a short 68nt NTR at 5′-terminus and an unusually long stretch of 406 nt NTR at 3′-terminal end. The protein sequences of P5 and P6 could not provide any information about the possible role they play in viral replication and have very low homology with viral proteins.

### Sequence diversity of RNA-5 in PPSMV-II isolates

**Figure 7:**
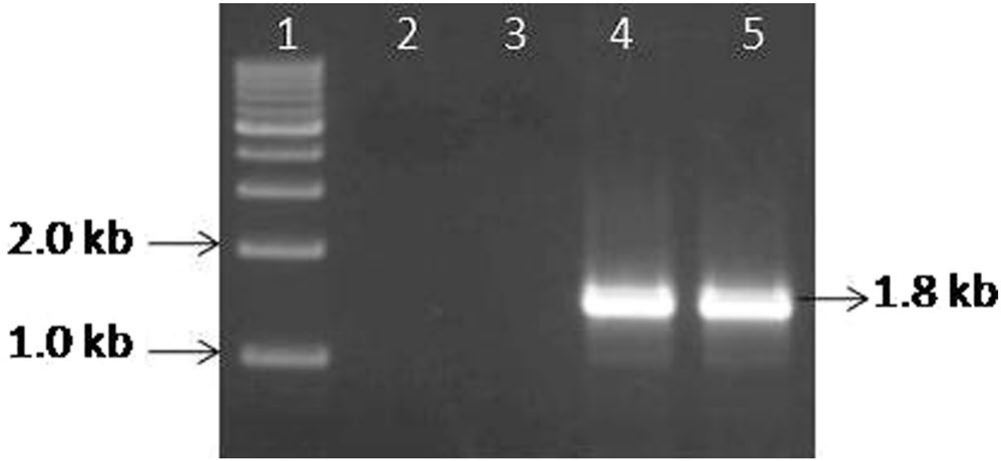
RT-PCR amplification of RNA-5 using specific primers synthesized from the 5′ and 3′-terminal ends. Standard DNA marker (lane M), healthy pigeonpea cv. ICP8863 (lane 1), pigeonpea cv. ICP8863 infected with PPSMV-I (sub-isolate Chevella) (lane 2), unknown pigeonpea cultivar infected with PPSMV-B isolate (lane 3) and pigeonpea cv. CO-5 cultivar infected with PPSMV-C isolate (lane 4). (The isolates “B” and “C” were found to contain only PPSMV-II). Specific amplification of 1.8 kb represents RNA-5 and was observed only in samples containing PPSMV-II.

Small RNA deep sequencing of infected pigeonpea cv. *Erra kandulu* cultivar (PPSMV-P sub-isolate Chevella) and Bng-1 sample (PPSMV-B isolate) generated contigs that revealed close identity with RNA-5. Sequence analysis confirmed a considerable variability among these contigs (Supplementary Table 3). RNA-5 amplified from PPSMV-P sub-isolate Chevella consisted of an amplicon of 1833 nt, whereas the amplified product from the PPSMV-B isolate (contains PPSMV-II with six genomic RNAs) is of 1812nt (Fig. 7). The variation in contigs was more pronounced near the 5′-and 3′ NTRs. While 5′and 3′-terminal farthest ends were entirely conserved in the RNA-5 of the two PPSMV isolates. PPSMV-II RNA-5 encodes 55 kDa protein consisting of 473 amino acids which could be confirmed in both PPSMV isolates with a 12% sequence divergence (Supplementary Table 3). Taken together the results suggest the possible existence of PPSMV-II strains in different locations. RNA-5 of PPSMV-2^10^ and PPSMV-II P sub-isolate Chevella being almost similar sharing 94.93% amino acid similarity. PPSMV-II RNA-5 showed low percent amino acid similarity with the orthologous proteins of other emaraviruses; RLBV (16.16), WMoV (19.02), AcCRaV (26.53) and a mere 32.77% similarity with closely related FMV, suggesting a high level of variability among RNA-5. However, we noticed that PPSMV-II RNA-5 had an exceptionally high similarity (86.47%) with PPSMV-1 RNA-5^9^. This anomaly can best be explained that PPSMV-1 so called RNA-5 might not relate to its genome. The degree of homology is too high to be expected (for RNA-5) between two different emaraviruses. Furthermore, PPSMV-I genome with four genomic RNA segments could be confirmed by the analysis of pigeonpea cv. ICP8863 plants infected by PPSMV-I alone (Fig. 7). Thus, it is possible that RNA-5 is associated with only one virus viz. PPSMV-II.

### Detection of PPSMV-I and PPSMV-II in SMD infected pigeonpea

RT-PCR with NCP primers, DIG-labelled probe and specific peptides (PPI and PPII) from both the viruses were synthesized and used to detect PPSMV-I and PPSMV-II employing RT-PCR, slot blot hybridization, and western blotting methods. The results of these experiments also confirmed the association of different PPSMVs with different isolates studied (Supplementary Table 4).

**Figure 8:**
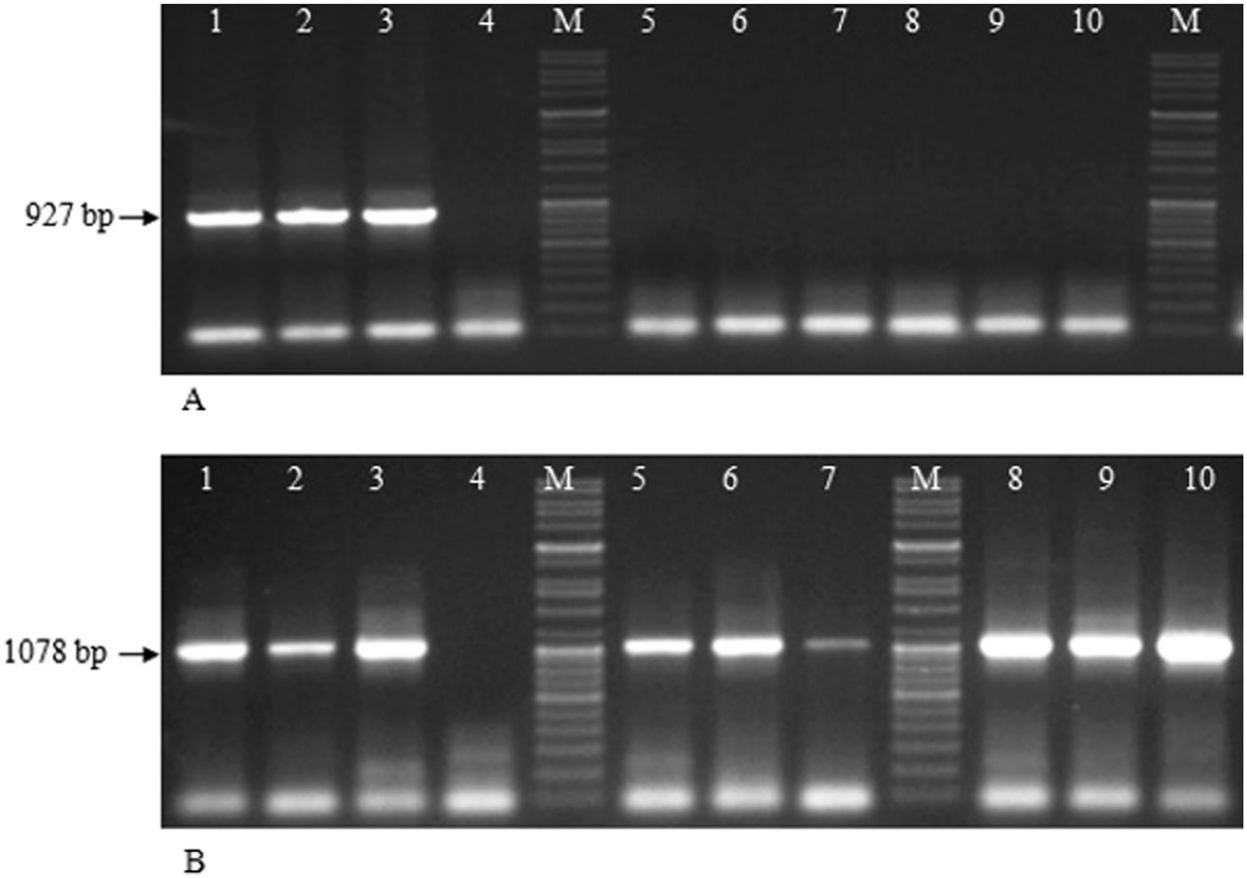
Resolution of RT-PCR (NCP-1 and NCP-2 primers) products in 1% agarose gels. Lanes contain PCR products from total nucleic acids used as template extracted from leaves of SMD affected pigeonpea cultivars. Mg-1 (lane 1), pigeonpea cv. ICP2376, (lane 2) pigeonpea cv. ICP8863 (lane 3) infected by PPSMV-P sub-isolate Chevella, total nucleic acids from healthy pigeonpea cv. *Erra kandulu* (lane 4); Bng-1 (lane 5), Bng-2 (lane 6), Bng-3 (lane 7), infected by PPSMV-B isolate; pigeonpea cv. CO-5 (lane 8), pigeonpea cv. CO-6 (lane 9) and pigeonpea cv. ICP8863 (lane 10) infected by PPSMV-C isolate; 5 kb DNA standard marker (lane M). (**A**) Detection of PPSMV-I; 927 bp amplified product related to the PPSMV-I NCP gene was detected only in plants collected from Chevella. (**B**)Detection of PPSMV-II; whereas 1078 bp amplified product related to the PPSMV-II NCP gene was detected in plants infected by PPSMV-P sub isolate-Chevella, PPSMV-B and PPSMV-C isolates (See supplementary Table-1).

RT-PCR analysis of SMD affected pigeonpea cultivars (*Erra kandulu*, ICP2376, ICP8863) infected with PPSMV-P sub-isolate Chevella revealed the presence of PPSMV-I by detecting specific amplicons (927 bp) of PPSMV-I NCP gene and PPSMV-II, by detecting specific amplicons (1078 bp) of PPSMV-II NCP gene. While PPSMV-II alone was detected in Bng-1, Bng-2, and Bng-3 from unknown pigeonpea cultivars infected with PPSMV-B and pigeonpea cvs. CO-5, CO-6, and ICP8863 infected with PPSMV-C isolates (Fig. 8). The presence of respective PPSMVs was confirmed by slot blot hybridization analysis (Fig. S10).

Riboprobes were synthesized from the most diverse regions of RNA-3 (nucleocapsid) of the PPSMVs (nucleotide position 103- to 492, NCP1 of PPSMV-I and 112- to 476, NCP2 of PPSMV-II) to ensure specific hybridization and used in the slot blot hybridization analysis. Each sample contained the equal amount of total nucleic acids (35 µg) applied to the respective slot on the blot. In this study, positive signals for detection of both PPSMVs NCP was detected only in plants infected by sub-isolate Chevella (Fig. S10, slots 1 and 2 of upper and lower rows in the blot), confirming the presence of both viruses in PPSMV-P sub-isolate Chevella, whereas nucleocapsid protein of PPSMV-II alone was detected in the plants infected by PPSMV-B (slots 4, 5, 6) and PPSMV-C (slots 7, 8, and 9) isolates. None of the non-symptomatic plants (slots 3, 10 and 11) showed the presence of PPSMVs (Fig. S10). Interestingly, RT-PCR analysis of mite inoculated (sub-isolate Chevella) pigeonpea cv. ICP8863 plants revealed the presence of PPSMV-I alone in some of the plants, while others showed the presence of both viruses. During our study routine mite inoculations resulted in few plants that carry only PPSMV-I. Western blot analysis using specific antiserum provided identical results confirming the distribution of PPSMV-I and PPSMV-II in the respective PPSMV isolates referred above. Total leaf proteins from the SMD affected pigeonpea cultivars from the three locations along with respective healthy plants were used in the immune-blotting experiment. The blots were probed with the antibodies (PPIAb and PPIIAb). The antibody detected a single 35 kDa protein in PPSMV-B and PPSMV-C infected plants and 34 kDa and 35 kDa proteins from plants infected by PPSMV-P sub-isolate Chevella (data not shown).

### Phylogenetic analysis

The phylogenetic trees developed showed emaraviruses clustered in two discrete clades. These two Emaravirus clades co-evolved with the members of *Orthobunyavirus*, and *Tospovirus Nairovirus*, *Tenuivirus* and *Phlebovirus* from a common ancestor (Fig. S9). Among the emaraviruses, one clade contains WMoV and RLBV while remaining seven emaraviruses are clustered into second clade with a common ancestor. PPSMV-I and PPSMV-II originate from a common ancestor branched to different clades, far away from each other and diversifying as two distinct viruses infecting a common host. Closely related PPSMV-II and FMV were present in the same clade reflecting evolutionary relationship, while PPSMV-I and RRV formed as closely associated taxa in a separate clade with a common ancestor, from other emaraviruses. The RNA-5 of PPSMV-II has phylogenetic relation with the emaraviruses and broadly in the pattern of RdRp, NCP and MP constituents. Phylogenetic tree of RNA-5 of PPSMVs was organized into a single clade with a common ancestor. The sister groups consisted of FMV and all variants of PPSMV-II as one clade, whereas the contentious PPSMV-1 though has a common ancestor branched to a different sub clade along with PPSMV-B isolate which contains only PPSMV-II, thus establishing relationship with PPSMV-II. RNA-5 sequences recently published (KP214012) evident from the analysis belongs to PPSMV-II group (Fig. S9).

Genome sequence of PPSMV-I and PPSMV-II have been submitted to GenBank under the following accession numbers. PPSMV-I: LN887951 (RNA-1), LN887952 (RNA-2), LN887953 (RNA-3) and LN887954 (RNA-4). PPSMV-II: LN651310 (RNA-1), LN651182 (RNA-2), LM652701 (RNA-3), LM652703 (RNA-4), LN898418 (RNA-5), LN898419 (RNA-6).

## Discussion

There was a major conundrum in etiology and molecular biology regarding the causal organism of SMD. In this report we tried to provide evidence of the association of an emaravirus (PPSMV-II) with the infected pigeonpea in three locations studied and co- existence of an additional emaravirus (PPSMV-I) with a smaller genome at one location. Initial analysis revealed that susceptible pigeonpea cv. ICP8863 showing typical SMD symptoms (infected by PPSMV-P sub-isolate Chevella) contain two non-homogeneous RNA-3 segments that encode nucleocapsid proteins and a single homogeneous RNA-3 component with PPSMV-B isolate infection. Interestingly we found doublets of the first four RNA segments subsequently with sub-isolate Chevella. The high-throughput small RNA sequencing facilitated to determine that the eriophyid mite (*Aceria cajani*) transmitted PPSMV-I containing four genomic RNAs and PPSMV-II containing six genomic RNAs. A single ORF is encoded by each genomic segment, which exists as a mixed infection in PPSMV-P sub-isolate Chevella, not found in any known plant diseases caused by emaraviruses and could be transmitted independently by eriophyid mite. However, plants infected by PPSMV-B and PPSMV-C isolates showed PPSMV-II alone. Pigeonpea cv. ICP2376, which produces severe mosaic with sterility symptoms when infected with PPSMV-B and PPSMV-C isolates^3^, produces chlorotic ring spots on the leaves with no sterility when inoculated with PPSMV-P sub isolate-Chevella (Fig. 1D–F). The presence of mixed infection of PPSMV-I and PPSMV-II in this isolate could be responsible for the symptom variation.

PPSMV-I genome with four RNA segments has modest sequence similarity with PPSMV-II orthologs and modest to weak similarity with the respective homologous proteins of known emaraviruses. The functional four RNA segments of both the viruses were identified based on sequence homology of other emaraviruses, encoding putative proteins identified as polymerase (P1), glycoprotein precursor (P2), nucleocapsid (P3) and movement proteins (P4), respectively. PPSMV-II RNA-5 and RNA-6 encoding P5 and P6 proteins, respectively are not related, with unknown functions. Interestingly RNA-1 to 4 of PPSMV-II has high sequence homology with FMV orthologs, and RNA-5 and RNA-6 have modest to weak homology with WoMV and RLBV orthologs. It is possible that the P5 to P6 proteins of PPSMV-II may be involved in the virus life cycle through different roles including transmission by eriophyid mites^14^.

The significant difference between our findings and the results of the previous investigation relates to composition of PPSMV-I genome. Elbeaino *et al*.^9^ described the PPSMV-1 genome containing an additional RNA (RNA-5). Conversely, we did not find hits specific to RNA-5 in the sequence analysis that could belong to PPSMV-I. Hits recognized in deep sequencing analysis, corresponding to RNA-5 were matched to PPSMV-II, could be confirmed by RT-PCR analysis using RNA-5 specific primers (Supplementary Table 5) indicating that PPSMV-1 may not have RNA-5 as genomic segment. Furthermore, few of the pigeonpea cv. ICP8863 plants showed PPSMV-I alone when inoculated with PPSMV-P sub-isolate Chevella instead of both the viruses. These plants did not show amplification of RNA-5 when specific primers were used (Fig. 7) confirming absence of the RNA-5. Phylogenetic relation of PPSMV-II RNA-5 with other emaraviruses including PPSMV-1 revealed that RNA-5 of PPSMVs formed single clade with a common ancestor (Fig. S9D). Taken together, it is possible that PPSMV-1 that was reported^9^ to contain five genomic RNAs is not convincing.

The segmented negative strand RNA viruses (sNSV) generally employ a ‘cap-snatching′ mechanism for viral mRNA transcription^39^ and are described well in *Bunyaviruses*, TSWV, and FMV^25,36,37^. Conserved sequence motifs and the domains associated with cap-snatching mechanism noticed in viruses of *Bunyaviridae* was identified in N-terminus of viral endonuclease subdomain (SRD-1) and probable thirteen conserved residues of cap-binding site in the central region of SRD-3 domains were identified in the RdRp of PPSMV-I and PPSMV-II (Fig. 3C; Table 3). The cap-binding sequences are not well conserved across the bunya viruses. However, PPSMV-II, FMV, and RRV had exact conservation. These findings facilitate to make a certain speculation that PPSMVs adopts a cap-snatching mechanism for viral transcription. This is the first known description of both the key constituents, may be involved in the mechanism have been identified in emaravirus RdRps. 3D structural analysis of endonuclease of PPSMVs and FMV revealed identical protein folding that is observed in Bunyaviruses and overall structural topology of these emaraviruses strikingly similar to *Bunyamwera virus* leading to functional similarity (Figs 5B and S6).

The amino acid sequence of central polymerase domain SRD-2 (Fig. 3C) of PPSMVs were aligned with viruses belonging to genera *Emaravirus*, *Tospovirus*, and *Bunyavirus*. The sequence alignment is primarily representing a conserved region of the palm sub-domain, the most highly conserved feature of all known polymerases^61^. Interestingly, 3D pol of PPSMVs (PPSMV-II shown here) resembles picornaviruses polymerase as closed right hand organization containing the conventional sub-domains, signifying the probable phylogenetic lineage though emaraviruses have negative strand RNA genome. 3D pol showing typical conserved sequence motifs A to E and motif F of the fingers region (Figs S4 and S5). Each motif is known to have a defined function for the synthesis of full length viral RNA^45^. Motif C of the viruses of the three genera (*Emara-*, *Tospo-*, and *Bunyavirus*) analyzed contains the catalytic center represented by sequence SDD (Ser1255-Asp1256-Asp1257 in PPSMV-II). The glycine residue of GDD motif, which is almost universally conserved in RdRps, has been found substituted by serine in the emaraviruses.

Plant viruses belonging to genera *Cytorhabdovirus*, *Nucleorhabdovirus*, *Tenuivirus*, *Tospovirus* and *Emaravirus* contain enveloped virus particles are known for the presence of glycoprotein-encoding ORFs^22^. The presence of the membrane glycoproteins plays a role in the viral entry into a host cell and engage in differential roles in protein folding and intracellular trafficking^62,63^. RNA-2 of PPSMVs encodes the glycoprotein precursor that is proteolytically cleaved to yield two viral structural glycoproteins; Gn and Gc, are known to be processed post-translationally. There is a modest amino acid sequence similarity (about 40%) of the precursor protein between PPSMVs and with other emaraviruses. RLBV and WMoV are the least comparable ones. The three-dimensional structure of the precursor protein with unique folding showing the characteristic features described previously. The precursor is shaped like a croissant with a panhandle (Fig. 6B) sharing several common features between PPSMVs (Table 3). The Gc substructure is loosely bound, ends in a panhandle containing the conserved motif of phleboviruses. This motif of eight residues is almost conserved among the emaraviruses compared to the members of genera of family *Bunyaviridae* with unknown function; however, it is speculated that phylogenetically, the Gc of the emaraviruses may be originated from Phleboviruses and the motif is an evolutional residual and be crucial for virus replication (Xiaohong Shi personal communication). 3D structures of the glycoprotein of FMV-JS1 and RRV were found identical with the PPSMVs (data not shown). The structural similarities suggest a common mode of activation for the above emaraviruses despite low sequence homology.

The putative nucleocapsid proteins (NCP) encoded by RNA-3 of the PPSMVs with a predicted size of 34.6 and 35.0 kDa, and the 3′ terminal ends of RNAs contain unusually long NTRs. The amino acid sequence of PPSMV-I NCP (34.6 kDa) has modest homology with PPSMV-II (35.0 kDa) and FMV, and weaker homology with the P3 proteins of other emaraviruses. Whereas PPSMV-II showed higher homology with the P3 of FMV (80.0%) and RRV (62.0%). The NCP share a set of conserved amino acid sequence motifs, NVLSFNK_134–140_, NRLA_183–186_ and GYEF_204–207_ with the other viruses of the genus *Emaravirus* as reported^14,16^, predicted to be associated with RNA-binding^27^ (Table 3). BindN analysis of NC protein of PPSMVs predicted stretch amino acid sequences at N′-terminal and C′-terminal regions also predicted to play a possible role in RNA binding^52^. In the present study, we determined the specificity of previously reported SMV-1 and SMV-2 primers^8^ generated from the PPSMV (Patancheru isolate) specific ‘32-kDa’ protein coding RNA to identify SMD. We noticed that these primers explicitly detect the PPSMV-I and not PPSMV-II in the PPSMV-P sub-isolate Chevella (Supplementary Table 1) and the tests were negative with pigeonpea plants infected by PPSMV-B and PPSMV-C isolates as they contain only PPSMV-II.

The causal agent requires a genomic entity for facilitating the spread of infection. The RNA-4 of both the PPSMVs encodes respective P4 proteins assumed to be involved in the cell-to-cell movement of the virus. McGavin, *et al*.^27^ provided evidence for localization of RLBV P4-GFP fusion protein in the cell wall and the plasmodesmata. Furthermore, emaraviral P4s are capable of rescuing the cell-to-cell movement of a defective *Potato virus X* by RLBV^53^ and FMV^54^ P4s, suggesting that P4 of these viruses are movement proteins and are interchangeable. The multiple sequence alignment of P4 of PPSMVs with other emaraviruses excluding the EMARaV, revealed their overall limited similarity apart from considerable conservation comprising hydrophobic amino acids in the central domain^57^ (Fig. S7). The secondary structure of PPSMVs of this region showed a similar configuration of series of α-helices and β-sheets identical to TMV 30 kDa movement protein^53,57^ (Table 3). PPSMVs contain two highly conserved regions of hydrophobic residues at the N-terminal end (Fig. S7) forms the secondary structure of two β-sheets (*β1* and *β2*) are identical to 30 K movement protein (Fig. S8)^53,55–57^. The 30 K domain containing these regions was described binding to the transported RNA and to translocate through the plasmodesmata^64^. Phylogenetic (Fig. S9C) and sequence analyses revealed that the P4 of WMoV and RLBV are distant members than the PPSMVs, FMV, RRV, RYRSaV and AcCRaV which have been recognized as the members of ‘30 K superfamily’ of MPs^19,53,54^. PPSMVs share several features with these emaraviruses may also belong to ‘30 K superfamily’ as well. P4 movement protein of PPSMVs contains amino acid sequences resemble DnaK protein may possess chaperone-like activity^55,65^ were identified in the C-terminal half of PPSMV-I and PPSMV-II (aa241–334 and aa239–336). However, the authenticity of this possible similarity has not been determined^53^. Secondary structures of PPSMVs in this region contain two long α-helices interconnected with long coils.

Although the first indication of the viral nature of SMD had emerged in 2003^8^, Koch’s postulates are yet to be fulfilled to recognize the sterility mosaic virus as causal organism of SMD. Clear association of viral RNAs especially PPSMV-II and its consistent presence in all locations studied, could help to rationalize that PPSMV-II could be the actual causal organism although this will be confirmed from the reaming locations. To hypothesize the co-evolution of PPSMVs in one host is still a question that needs to be addressed along with relationship between them in terms of pathogenesis with more experimentation. The identification of these diverse viruses provides a new perspective on the evolutionary origins of multipartite viruses with negative-sense RNAs. In particular, PPSMV-I that is different from PPSMV-II, occupying a phylogenetic position with RRV, RYSaV and EMARaV, containing genomes with four RNA components.

## Materials and Methods

### Virus isolates and nomenclature

Several PPSMV isolates have been identified at different locations in India based on genotype reaction and were grouped in to five variants^3^. The same nomenclature was used to describe PPSMV isolate, at a given location^66^. These variants are referred in general terms as isolates; PPSMV-P (Patancheru, Telangana state), PPSMV-B (Bengaluru, Karnataka state) and PPSMV-C (Coimbatore, Tamil Nadu state). In addition to Bengaluru and Coimbatore locations, we collected SMD affected leaf samples from Chevella village area (Telangana state), 45 to 50 kilometers away from Patancheru. For sample collection, no specific permission was required. Our study revealed that plants from Chevella were infected with PPSMV-P isolate that contains PPSMV-I and PPSMV-II, named as PPSMV-P sub-isolate Chevella (Supplementary Table 4).

### Sterility mosaic infected plants collection

We surveyed the three above mentioned locations and collected infected leaf samples. Pigeonpea plants (Pigeonpea cv. *Erra kandulu*) with typical SMD symptoms were identified, and young leaves were collected. The infected samples were labelled as Mg-1, Mg-2, and Alr representing the three pigeonpea fields. Similarly, second set of samples was collected from Karnataka state around University of agriculture sciences, GKVK campus. Infected leaf samples (PPSMV-B isolate) from Bng-1 (Rajankunte location), Bng-2 (Singhnarayanhalli farm) and Bng-3 (UAS) were collected as unknown pigeonpea local cultivars. The third set of infected samples was collected from Tamil Nadu Agriculture University (TNAU) fields located at Coimbatore (Supplementary Table 4). In TNAU, PPSMV-C isolate is maintained in pigeonpea cvs. CO-5, CO-6 and ICP8863 in the fields. Samples were collected from these infected cultivars. Samples were collected as a pool of the same cultivar from each field along with plants showing no symptoms as healthy controls. Leaf samples were plucked in labelled separate Ziploc bags placed in ice chest with dry ice and transported to the lab. SMD infected pigeonpea samples from the field may be infected by other viruses as low and rare incidences of other viral infections^67,68^ have been reported. In addition, *Tobacco streak virus* (TSV) has been reported affecting pigeonpea plants in the fields^69^.

### Maintenance of virus culture and production of infected plants

PPSMV-P sub-isolate Chevella was maintained in pigeonpea cvs. ICP8863 and ICP2376. Virus transmission was done by using young SMD infected pigeon pea leaflets infested with mites, stapled to the primary leaves^70^ of pigeonpea seedlings (Fig. S1). Stapled plants were kept in insect proof glass house (average 28 °C day and 24 °C night) and symptoms were observed.

### Isolation of dsRNA and total RNA from SMD affected leaves

Pigeonpea leaves with mosaic mottling symptoms and non-symptomatic leaf samples as controls of each location, collected from Chevella (Mg-1, Mg-2, and Alr), Bengaluru (Bng-1, Bng-2 and Bng-3) and Coimbatore (pigeonpea cvs. CO-5, CO-6 and ICP8863) as well as mite inoculated pigeonpea cvs. ICP8863 and ICP2376 (35 dpi) were used to isolate dsRNA as described^71^. Isolated dsRNA was fractionated on an agarose gel (1%) containing ethidium bromide (10 mg/ml) and visualized in gel doc system (Syngene, UK). *Cucumber mosaic virus* (CMV) dsRNA, extracted from *N*. *tabacum* cv Xanthi was used as dsRNA size marker. Total nucleic acid from infected leaves collected from the three locations and mite inoculated (ICP886 and ICP2376, 35 dpi) were isolated by CTAB method^72^. The quantity of RNA obtained was estimated by spectrophotometer (Nanodrop 1000, Thermo Scientific Waltham, MA, USA). The protocol resulted in the isolation of 10–20 µg RNA/150 mg of leaf sample.

### Viral detection by using SMV primers–RT-PCR analysis

SMV-1 and SMV-2 oligonucleotide primers (known for the amplification of a 321-bp product) were developed from partial nucleocapsid sequence (764 bp; AJ439561) of PPSMV-P^8^ to determine the specificity of these primers to PPSMV-I and PPSMV-II. About 1 µg of total RNA from SMD affected Mg-1, Mg-2, Bng-1, Bng-2, Bng-3, CO-5 CO-6 and pigeonpea cv. ICP8863 and mite inoculated ICP8863 (PPSMV-P sub-isolate Chevella) leaf tissues were used as template for the assay. The protocols for RT reaction, first strand cDNA synthesis and polymerase chain reaction of the primers were followed as described^8^. Total RNA from the non-symptomatic pigeonpea cv. ICP8863 and unknown cultivar (Bengaluru) from the three locations were used as control. RT-PCR amplicons were fractionated on 1% agarose gel containing ethidium bromide and visualized in a gel doc (Syngene, UK).

### Small RNA extraction, library preparation, and Illumina sequencing

Small RNA population was separated from total RNA extracted from SMD affected leaves of Mg-1, Mg-2 (from Chevella) and Bng-1 (from Bangaluru) by CTAB method^72^. Total RNA (7 µg) was fractionated on 12% polyacrylamide gel electrophoresis (PAGE), and the RNA molecules with size ranging 18–30 nts were eluted from the gel. This small RNA population (50 ng) was used for library preparation (after QC analysis on Bioanalyzer) by using NEXTflex small RNA library preparation kit V3 (Bioo Scientific, Austin, TX, USA) at Genotypic Technology genomics facility, Bengaluru. The procedure (in brief) for library preparation was as follows: adapters were ligated at 3′ and 5′ ends of the purified small RNAs. Ligated RNAs were reverse transcribed, and the resulting cDNA library was enriched by PCR (12 cycles). Further resolution, size selection (140–160 bp) of enriched cDNA library was done on 8% PAGE and the fragments were eluted from the gel. The prepared library was quantified by using Qubit fluorometer (Agilent, USA) and the quality was determined by using high sensitivity Bioanalyzer chip (Agilent, USA). sRNA (10 nM) enriched fraction was used for single end read sequencing on Illumina GAIIx platform.

### Sequence analysis

To obtain high-quality reads, total reads were filtered by SeqQC-V2.0 program (http://genotypic.co. in/SeqQC.html). Adapters were trimmed from the filtered reads used for subsequence assembly. To enrich virus derived small interfering RNA (vd-siRNA) pool, the obtained reads were mapped on pigeonpea genome to remove host specific sRNAs by using Bowtie 2.1.0 program^73^. Filtered unaligned reads were used for generation of contigs. *De novo* assembly of short reads was performed by using Velvet -1.2.10 based on de Bruijn graph^74^; with k-mer17 (often we noticed large k-mer resulting high specificity in contigs). For generation of contigs corresponding to viral genome siRNA reads 20–22 bp length were used for viral genome assembly. The identity of these contigs was established by Blast and BlastX search in NCBI database (Supplementary Table 6).

### Validation of contigs identity

Based on obtained sequence information, primers were designed (Supplementary Table 5). Specific primers were used for first strand cDNA synthesis by reverse transcription (RT) and polymerase chain reaction (PCR). For first strand cDNA synthesis, 1 µg RNA of SMD affected pigeonpea samples were used for RT reaction. RNA was denatured at 65 °C for 5 min and immediately chilled on ice. RT reaction mixture containing 1x RT buffer, 1 µl dNTPs (10 mM each), 1 µl of specific reverse primer (10 pmol), 1 µl (200 units) of Reverse Transcriptase (Primescript, TakaraBio, Japan) was added to the denatured RNA and the final volume was adjusted to 25 µl with nuclease free water. The RT reaction mixture was incubated at 42 °C for one hr and then 70 °C for 15 min to deactivate the enzyme. The cDNA obtained was used directly for PCR using gene specific primers in 50 µl reaction volume. PCR reaction contained 3.0 µl of cDNA, 1.0 µl of 2.5 mM each dNTP mixture, 1x GC melting buffer, 1.0 µl of 10pmole primer, 0.2 µl (1unit) Taq DNA polymerase (GC LA Taq, TakaraBio, Japan) and the final volume was adjusted with nuclease free water. Amplicons were fractionated on 1% agarose gel containing ethidium bromide and documented by GBox EF2 Geldoc system (Syngene, UK). Specific amplicons were excised from agarose gel, eluted, cloned in pGem-T easy vector (Promega, USA) and sequenced by Sanger’s dideoxy chain termination method. ClustalW program was used for multiple alignments of protein sequences of respective genes of PPSMVs and percent amino acid homology with all emaraviruses related sequences were determined^75^.

### Phylogenetic analysis

To examine the phylogenetic relationship of PPSMVs with related viruses, amino acid sequences of RdRp (P1), NCP (P3) and MP (P4) of PPSMV-I and additional RNA-5 (P5) of PPSMV-II and the orthologs of known emaraviruses were aligned with viral proteins of representative members of family *Bunyaviridae* and genus *Tenuivirus*, and used in constructing the phylogenetic trees. The viruses used in the analysis are as follows: genus *Emaravirus*: EMARaV, FMV, PPSMVs, RRV, RLBV, RYRSaV, WMoV and AcCRaV; genus *Orthobunyavirus*: *Bunyamwera virus* (BUNV) *La Crosse virus* (LACV); genus *Tospovirus:* represented by *Tomato spotted wilt virus* (TSWV) and *Groundnut bud necrosis virus* (GBNV); genus *Hantavirus*: *Hantaan virus* (HTNV), *Puumala virus* (PUUV); genus *Tenuvirus*: *Rice streak virus* (RSV), *Rice grassy stunt virus* (RGSV) genus: *Nairovirus*: *Dugbe virus* (DUGV), *Crimean-Congo hemorrhagic fever virus* (CCHFV). Multiple alignments of protein sequences were performed using ClustalW^75^. Phylogenetic trees were constructed by MEGA6.0^76^; using neighbour-joining method^77^ employing maximum likelihood (ML) criterion with 1000 bootstrap replicates, to examine the evolutionary history and evolutionary distance^78^.

### Synthesis of nucleocapsid peptides for production of antibodies

#### Synthesis of nucleocapsid peptides

The putative nucleocapsid proteins (NCP) of PPSMV-I and PPSMV-II have a close similarity in amino acid sequence. However, we identified specific unique peptide sequences of both the nucleocapsid proteins to generate antisera. Peptide sequences specific to nucleocapsid of PPSMV-I (PPI_5–20_ MPSKTPFSNMPAASKK*) and PPSMV-II (PPII_299–314_ *STFLPALEADRLASLP) were synthesized. Peptides were synthesized from GL Biochem limited (Shanghai, China). These peptides were conjugated to keyhole limpet hemocyanin (KLH) at cysteine residue i.e. “*” position which acts as a carrier. The antisera (PPIAb and PPIIAb) were used to detect the specific viruses.

#### Production of antibodies

PPI and PPII synthetic peptides thus developed of PPSMV-I and PPSMV-II were used as an antigen to generate respective antisera using New Zealander white albino rabbits. Pre-immune bleed of 2 ml per rabbit as control serum was collected on day 0, followed by the primary injection. Two hundred microgram (200 µg) of peptide-KLH conjugates emulsified with Complete Freund’s Adjuvant (CFA) (Merck Biosciences, Germany) was injected as a primary injection, to eight-week old rabbits by intramuscular injections^79,80^. Subsequently, three booster injections of 300, 400 and 500 µg of KLH-peptide conjugate antigen, emulsified with Incomplete Freund’s Adjuvant (IFA) were injected at two-week intervals. After ten days of the third booster injection, the animal was bled, and antiserum was collected. IgG fraction from the crude antibody sera was purified by IgG purification kit (Merck Biosciences, Germany) following manufacturer instructions. The purified antibodies (PPIAb and PPIIAb) were used for the detection of specific PPSMVs by western blotting. For raising antisera, rabbits were used after approval from the Institutional Animal Ethics Committee under Committee for the purpose of control and supervision on experiment on animals (CPCSEA). All the experiments were conducted as per guidelines and regulations of the committee.

### Structural analysis of viral endonuclease, glycoprotein, and movement protein

The three-dimensional structures of polymerase (RdRp, P1) and glycoprotein precursor (P2) proteins of PPSMV-I and PPSMV-II were studied. The conserved amino acid sequence of viral polymerase at N-terminal SRD-1 domain of P1 (amino acid position 66 to 242) and glycoprotein precursor P2 (PPSMV-I 648 amino acids and PPSMV-II 649 amino acids) proteins were used to develop 3D structures with their characteristic folding. I-TASSER, (http://zhanglab.ccmb.med.umich.edu/I-TASSER), on line server was used to study the proteins^47,81^ in their tertiary form. The secondary structures of P4 ‘movement protein’ were predicted using PSIPRED server^82^ (http://bioinf.cs.ucl.ac.uk/psipred/). The outputs of the I-TASSER data of possible predicted models and the molecular graphics are of high quality. These contained full-length secondary and tertiary structure predictions and was subjected to simulations by an open-source viewer, the PyMol (https://www.pymol.org ) and Jmol (http://jmol.sourceforge.net), an interactive graphics program^83,84^, for illustrating the three-dimensional chemical structures. These programs facilitated to alter the scheme of the images and to characterize these proteins by identifying conserved residues and the motifs in the protein folding.

### Ethical approval and informed consent

For raising antisera rabbits were used after approval from the Institutional Animal Ethics Committee under Committee for the purpose of control and supervision on experiment on animals (CPCSEA).

## Electronic supplementary material

Supplementary information 
