# Supplementary material for: Molecular characterization of emaraviruses associated with Pigeonpea sterility mosaic disease
Source: Sci Rep. 2017 Sep 19;7:11831. doi: 10.1038/s41598-017-11958-8 (PMC5605523; doi:10.1038/s41598-017-11958-8)
Supplement: Supplementary file 1 — Supplementary information [file 41598_2017_11958_MOESM1_ESM.doc]

**Molecular characterization of emaraviruses associated with Pigeonpea sterility mosaic disease**

Surender Kumar^1, 2^, BL Subbarao^3^, Vipin Hallan^1, 2^ *

**Supplementary Information**

**Supplementary Figures**

**Fig. S1.** Leaf stapling inoculation of PPSMV-P sub-isolate Chevella. 12 days old pigeonpea cv. ICP8863 seedlings stapled with SMD affected pigeonpea leaflets carrying viruliferous mites. This simple and efficient inoculation technique when inoculated susceptible cultivars normally show 100% infection. Typical initial symptoms of SMD can be seen in 10 to 15 dpi. To generate more infected material for quick extractions we used inoculating bunch of seedlings at times, otherwise it would always single plant inoculations were adopted.

**Fig. S2.** Electrophoretic separation in 1% agarose gel of dsRNA isolated from SMD affected pigeonpea cultivars infected by Coimbatore and Bangaluru isolates: dsRNA extracts of non symptomatic pigeonpea cv. ICP8863 (lane1), dsRNA from SMD affected pigeonpea cv. ICP8863 infected by PPSMV-C isolate (lanes2), and Bng-1 of unknown pigeonpea cultivar infected by PPSMV-B isolate (lanes 3), dsRNA from a random infected pigeonpea plant showing symptoms different from SMD (Lane 4), Healthy unknown pigeonpea cultivar (lane 5) Lane, M-standard DNA marker (Takara Bio, Japan). *Note*: The PPSMV-II six dsRNAs labelled on the left pointing to the lanes 2 and 3.

**Fig. S3** Resolution of RT-PCR amplified product in 1% agarose gel. Total nucleic acids as template isolated from pigeonpea cultivars infected by PPSMV isolates and SMV-1and SMV-2 primers were used in the amplification. Lane-M Standard 1kb DNA marker (Takara, Japan), lanes 1-3 containing PCR product (321bp) from Mg-1, Mg-2 pigeonpea cv. (*Erra kandulu*), and mite inoculated pigeonpea cv.ICP8863 (infected by PPSMV-P sub-isolate Chevella). Lanes 5-7 contain Bng-1 Bng-2 and Bng-3 (pigeonpea unknown cultivar) infected by PPSMV-B isolate and lanes 9-11 contain pigeonpea cv.CO-5, pigeonpea cv.CO-6 and pigeonpea cv.ICP8863 cultivars infected by PPSMV-C isolate respectively. Lanes 4 (ICP8863), 8 (non symptomatic pigeonpea cultivar) and 12 (ICP8863) contain respective healthy controls from the three locations.

**Fig. S4** Cartoon representation of the PPSMV-II 3Dpol structure (at an ave. 9.2 Å resolution; front view). The 3D pol contains the conventional sub domains as fingers, palm (catalytic center) and thumb showing the typical sequence motifs. The six conserved structure-sequence motifs are colored: A, pale blue; B, brown; C, magenta; D, orange; E, red and F, blue identified in the polymerase. 3D structure was developed using RdRp sequence (between residues 652 and 1400) of SRD-2 domain, commonly referred as Bunya RdRp region of RNA-1 (Fig. 3C).

**Fig. S5 A** Multiple amino acid sequence alignment of endonuclease domain showing presence of conserved amino acid (H....D...PD....DxK) residues. Conserved residues were shown in red letters.

**Fig. S5 B** Amino acid sequence alignment of Bunya RdRp like central region (1025-1332) of SRD-2 domain of PPSMV-I, RNA dependent RNA polymerases (RdRp) with PPSMVII, FMV-JS1, EMARaV (*Emaravirus)*, TSWV, GBNV (*Tospovirus*) and BunyaV (*Orthobunyavirus*). Functional motifs A, B, C, D, E and F were identified in the aligned sequences**.** Individual active residues and of the conserved motifs are indicated with red, more than four individual conserved amino acids in a row are coloured blue.

**Fig. S6.** Three dimensional structure of endonuclease of *Bunyamwera virus* (NC_001925.1) was resolved at 1.3Å. The endonuclease contains a typical folding pattern with seven α-helices and four β-sheets. The predicted overall topology of PPSMV-I and PPSMV-II endonuclease is similar to bunya endonuclease structure. Universally conserved motifs RHD (green), TPD (orange) and motif DYK (purple), and the active residues T105, Y109, I128, P133 (cyan) associated were indicated

**Fig. S7** Multiple alignment of amino acid sequence representing the central region of movement proteins of seven emaraviruses. The central part of emaravirus P4s that is similar to TMV 30kDa movement protein, starts with the LPI motif (I-69) and ends with motif SWKT (T-199in PPSMV-II) is indicated. Conserved regions of aliphatic residues (V/I, β-1) and the consensus aspartate in DxR motif (D, β-2) have been highlighted. The sequences in the central region containing 40 amino acid (TAV segment) shown to contain conserved motifs shared homology with first six viruses. In addition there are several small residues aligned are conserved. The alignment pattern in general indicates that RLBV and WMoV are distantly similar to the 30K than the PPSMVs, FMV, RRV, RYRSaV and AcCRaV. Six or all residues in a row and peptides (motifs) of the PPSMVs aligned with other emaraviruses were coloured red. Other conserved residues were coloured blue. EMARaV was not included as the accessions are partial sequences (YP_003104766; CCH80657.1)

**Fig. S8** Alignment of β-1 and β-2 region consensus sequences of ‘30K’ (TMV) and the movement protein superfamily members of (*Bunyaviridae*) genera of *Tospovirus* (TSWV) and *Tenuivirus* (RSV) with the members of genus *Emaravirus*. Consensus sequences indicating hydrophobic ( ) and aliphatic ( ) residues of β sheet 1 were identified. Absolutely conserved aspartate D113 (in PPSMV-II) of β sheet 2 and the hydrophobic residues have been indicated. The nomenclature of 30K MPs secondary structure elements has been used. FMV, RRV, RYRSaV, RLBV and AcCRaV have been identified as ‘30K’ superfamily possible members.

**Fig. S9** Phylogenetic analyses of emaraviruses, and viruses belonging to genera of *Bunyaviridae*, and *Tenuivirus*. Phylogenetic trees were constructed using amino acid sequences of RdRp (A), NCP (B) P4 (C) and P5 (D) by MEGA 6.0 using neighbour joining method. Evolutionary history and evolutionary distances were inferred using the maximum likelihood (ML) criterion with 1000 bootstrap value. PPSMV-II and FMV were present in the same clade while PPSMV-I and RRV constituted a closely associated taxon in a separate clade with a common ancestor. Phylogenetic relationship of P5 (RNA-5) of PPSMV-II variants present in a single clade along with FMV whereas the contentious PPSMV-1 (RNA-5) though has a common ancestor branched to a different sub clade along with PPSMV-B isolate which contains only PPSMV-II, thus establishing relationship. The bar represents the number of amino acid changes per site.

**Fig. S10** Slot blot hybridization analysis of different PPSMV isolates: Total nucleic acids as template isolated from pigeonpea cultivars infected by PPSMV isolates probed with RNA-3 (NCP-1 and NCP-2 primers) from PPSMV-I (A) and PPSMV-II (B). Mg-1 (slot-1), Mg-2 (slot -2) infected by PPSMV-P sub-isolate Chevella; Bng-1(slot -4), Bng-2 (slot-5) and Bng-3 (slot-6) infected by PPSMV-B isolate; pigeonpea cv. ICP-8863(slot-7) pigeonpea cv. CO-5 (slot-8) and pigeonpea cv. CO-6 (slot-9) cultivars infected by PPSMV-C isolate. Healthy pigeonpea cv. *Erra kandulu* (slot-3), pigeonpea unknown cultivar (slot-10) and pigeonpea cv. ICP-8863 (slot-11) were used as controls.

**
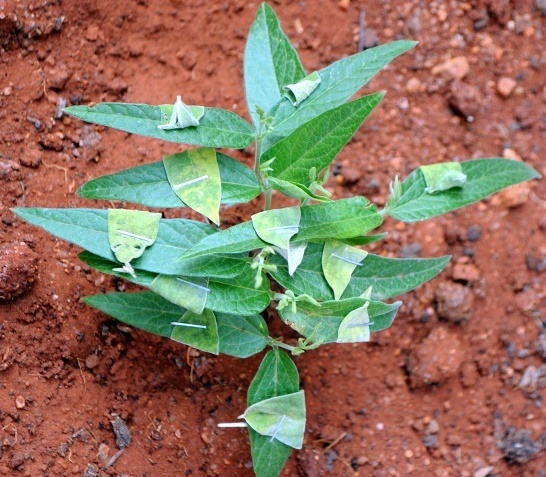
**

**Fig. S1 (Hallan)**

**
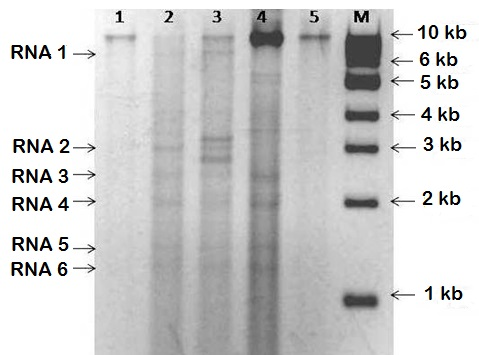
**

**Fig. S2 (Hallan)**


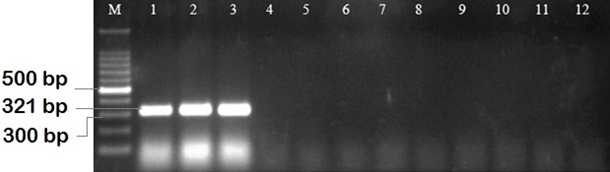


**Fig. S3 (Hallan)**

**
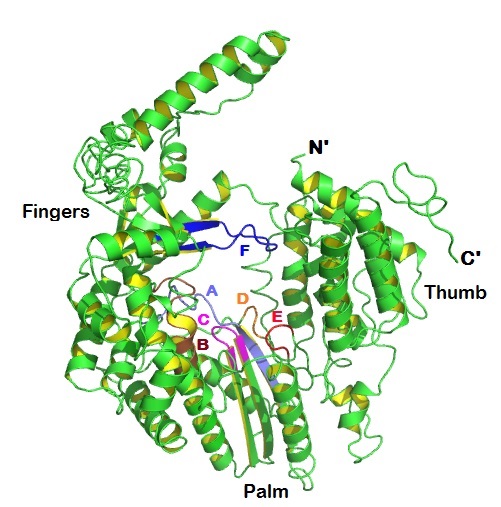
**

**Fig. S4 (Hallan)**

**
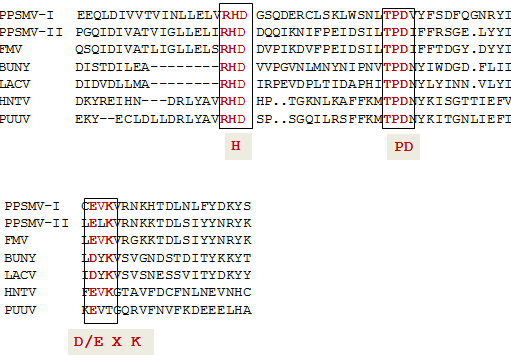
**

**Fig. S5A (Hallan)**

**
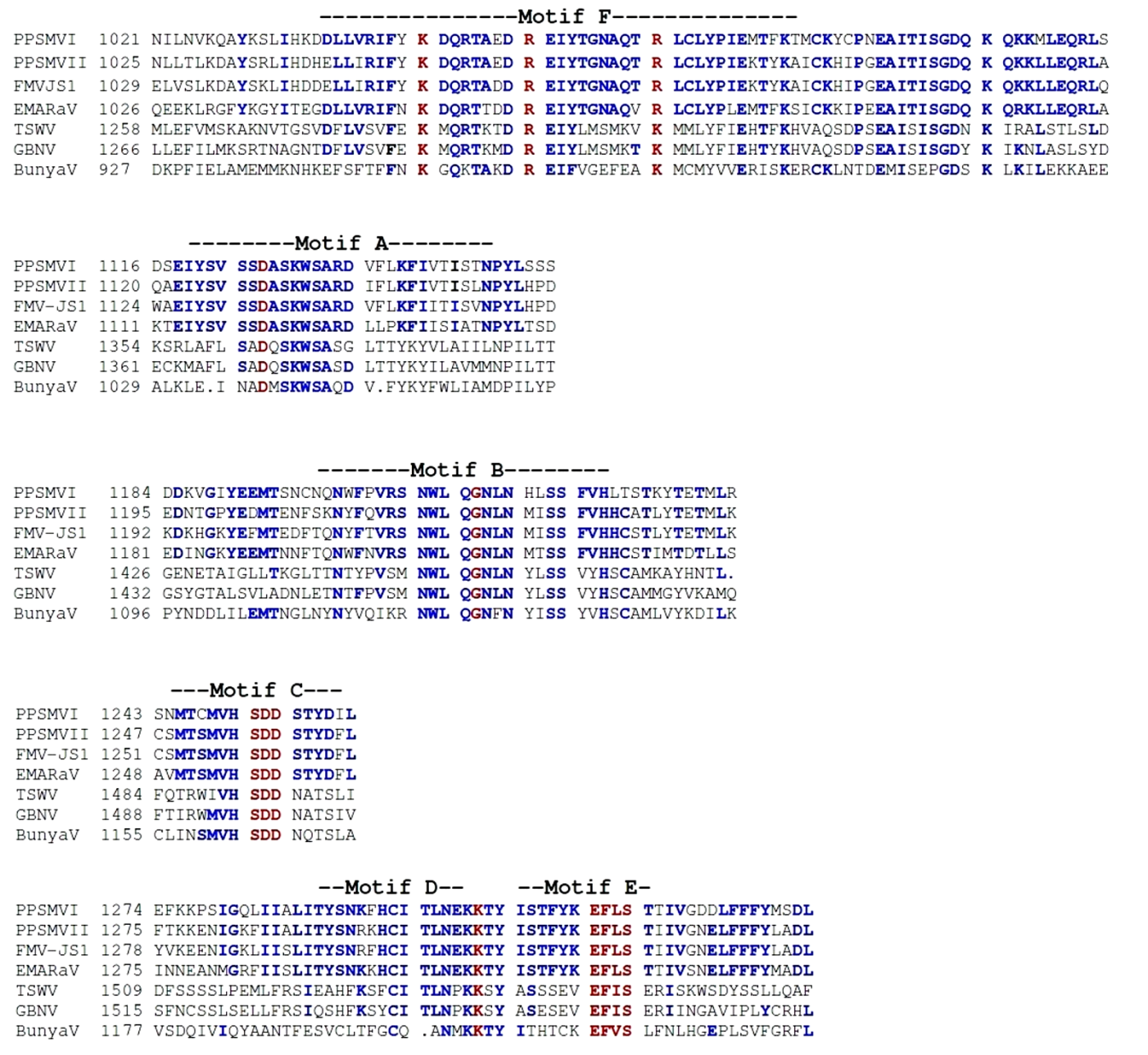
**

**Fig. S5B (Hallan)**

**
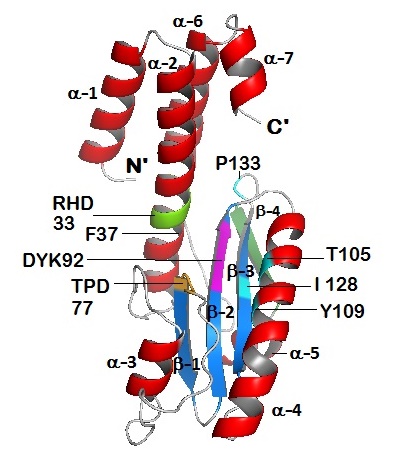
**

**Fig. S6 (Hallan)**

**
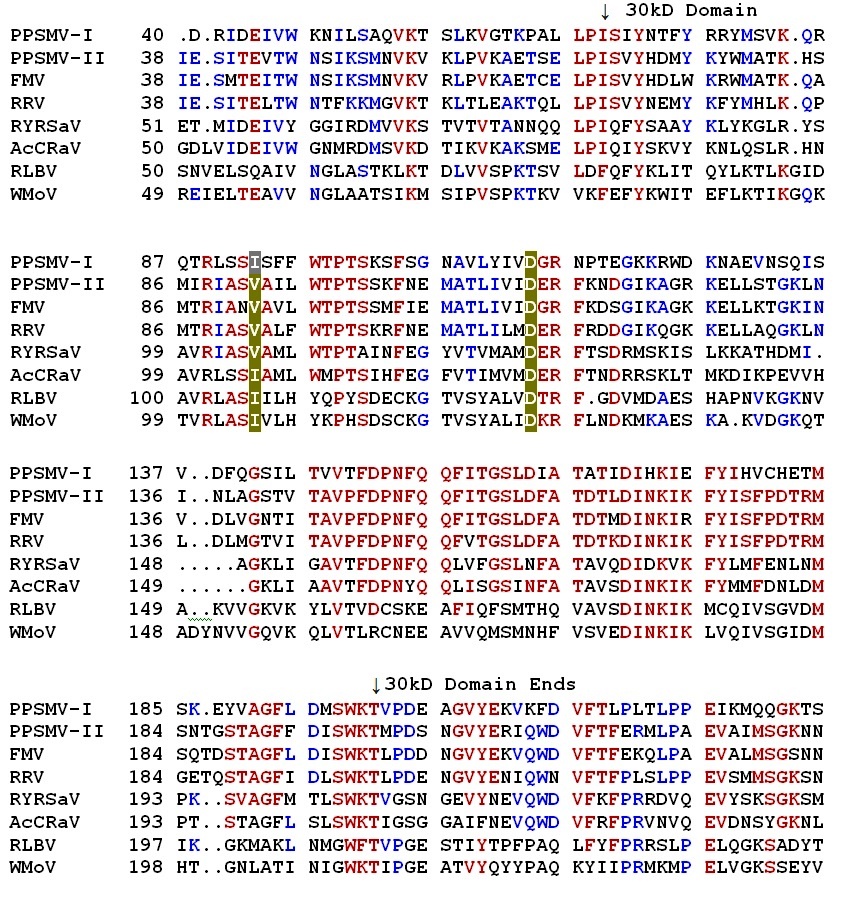
**

**Fig. S7 (Hallan)**


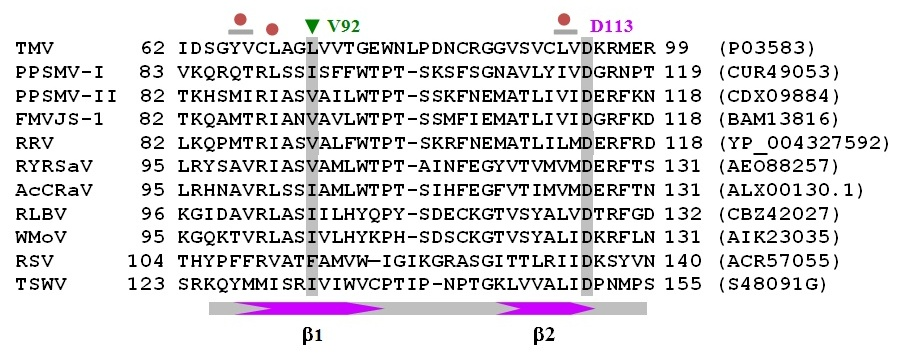


**Fig. S8 (Hallan)**

**
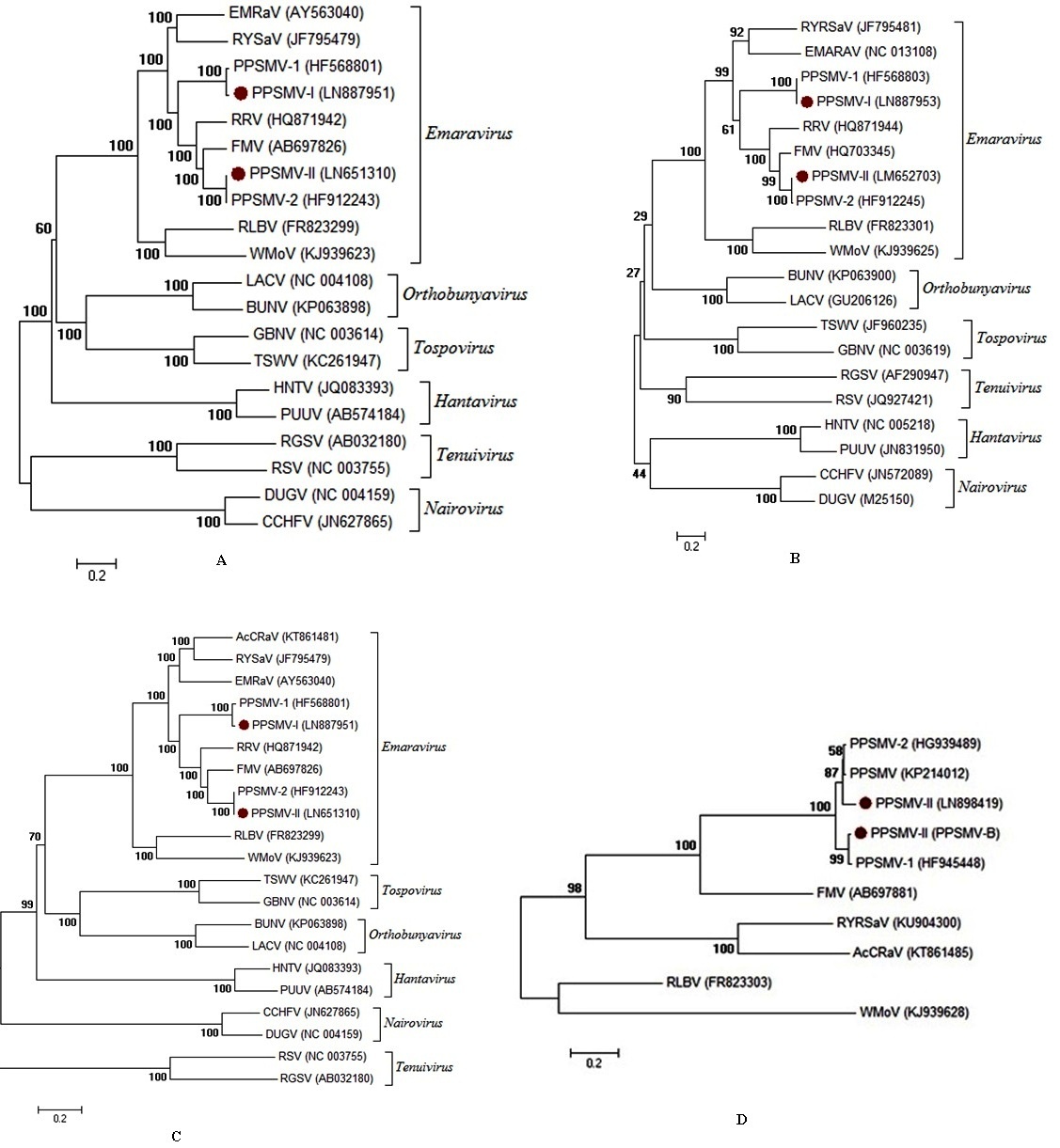
**

**Fig. S9 (Hallan)**

**
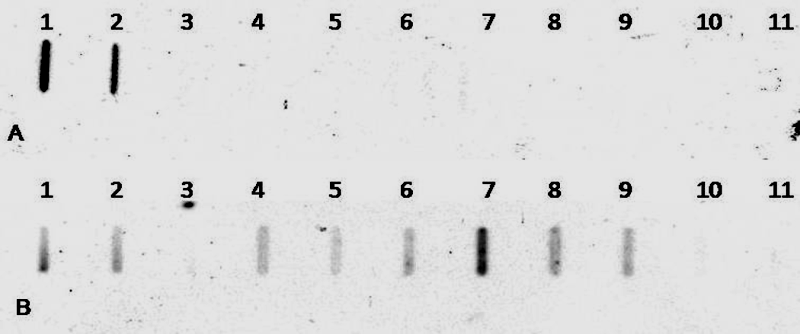
**

**Fig. S10 (Hallan)**

**Supplementary Tables**

**Supplementary Table 1:** List of primers used for the detection of PPSMV-I and PPSMV-II

**Supplementary Table 2:** Distribution of RNA contigs found in high-throughput sequencing of SMD affected Mg-1 and Mg-2 samples (PPSMV-P sub isolate-Chevella) and in PPSMV-B isolate.

**Supplementary Table 3:** Comparison of percent amino acid similarity of RNA-5 in PPSMVs

**Supplementary Table 4:** Sterility mosaic disease (SMD) affected pigeonpea collected from different locations in India, infected by distinct PPSMV isolates

**Supplementary Table 5:** List of primers used for genome amplification and sequencing of PPSMV-I and PPSMV-II

**Supplementary Table 6:** Table showing details of number of reads and their quality analysis

**Supplementary Table 1:** List of primers used for the detection of PPSMV-I and PPSMV-II

| **Primer name** | **Virus** | **Primer sequence**  **(5’ - 3’)** | **Target region and amplicon size (bp)** |
| --- | --- | --- | --- |
| CP1FP | PPSMV-I | CAATGCCTCCAAAGATGCCATCTAAAACT | NCP; 927 |
| CP1RP | PPSMV-I | TTACTCCTTTAAAGATTTCAAAAGCTCTTC |
| CP2FP | PPSMV-II | ACCTGGTGTTTATTCCTCTCAAAGGCAC | NCP; 1078 |
| CP2RP | PPSMV-II | TACAGTCCGACGATCATAGAACACACC |
| SMV-1 | PPSMV* | ACATAGTTCAATCCTTGAGTGCG | NCP; 321 |
| SMV-2 | PPSMV* | ATATTTTAATACACTGATAGGA |

PPSMV*: Sterility mosaic pathogen was named as PPSMV before the discovery of mixture of viruses related to Patancheru isolate. (In this study we found SMV primers are related to PPSMV-I)

**Supplementary Table 2:** Distribution of RNA contigs found in high-throughput sequencing of SMD affected Mg-1, Mg-2 (PPSMV-P sub isolate-Chevella) and in Bng-1 (PPSMV-B isolate) samples.

| **Contig number** | **Sequence length (bp)** | **Characters of reads/contigs**  **(Genomic segments)** | **Viral homology**  **(BlastX)** |
| --- | --- | --- | --- |
| **Mg-1** |  |  |  |
| 1 | 2111 | RNA-2 (GP) | PPSMV-II |
| 4 | 1265 | RNA-4 (MP) | PPSMV-I |
| 5 | 1150 | RNA-3 (NCP) | PPSMV-I |
| 8 | 1007 | RNA-3 (NCP) | PPSMV-II |
| 14 | 858 | RNA-5 | PPSMV-II |
| 16 | 696 | RNA-4 (MP) | PPSMV-II |
| 20 | 598 | RNA-6 | PPSMV-II |
| 34 | 421 | RNA-1 (RdRp) | PPSMV-II |
| 35 | 420 | RNA-2 (GP) | PPSMV-I |
| 47 | 353 | RNA-1 (RdRp) | PPSMV-II |
| 53 | 324 | RNA-4 (MP) | PPSMV-II |
| 56 | 318 | RNA-1 (RdRp) | PPSMV-II |
| 60 | 305 | RNA-1 (RdRp) | PPSMV-II |
| 61 | 299 | RNA-2 (GP) | PPSMV-I |
| 62 | 291 | RNA-2 (GP) | PPSMV-I |
| 64 | 288 | RNA-3 (NCP) | PPSMV-II |
| 68 | 279 | RNA-1 (RdRp) | PPSMV-II |
| 74 | 272 | RNA-2 (GP) | PPSMV-I |
| 75 | 271 | RNA-2 (GP) | PPSMV-I |
| 76 | 269 | RNA-1 (RdRp) | PPSMV-II |
| 107 | 217 | RNA-1 (RdRp) | PPSMV-II |
| 114 | 210 | RNA-1 (RdRp) | PPSMV-II |
| 125 | 197 | RNA-1 (RdRp) | PPSMV-II |
| **Mg-2** |  |  |  |
| 1 | 5822 | RNA-1 (RdRp) | PPSMV-I |
| 2 | 2976 | RNA-2 (GP) | PPSMV-I |
| 3 | 2500 | RNA-4 (MP) | PPSMV-II |
| 4 | 1833 | RNA-5 | PPSMV-II |
| 5 | 1766 | RNA-1 (RdRp) | PPSMV-II |
| 10 | 1264 | RNA-1 (RdRp) | PPSMV-I |
| 11 | 1255 | RNA-1 (RdRp) | PPSMV-II |
| 13 | 1222 | RNA-4 (MP) | PPSMV-I |
| 14 | 1194 | RNA-6 | PPSMV-II |
| 15 | 1172 | RNA-2 (GP) | PPSMV-I |
| 19 | 909 | RNA-3 (NCP) | PPSMV-I |
| 24 | 814 | RNA-1 (RdRp) | PPSMV-II |
| 29 | 757 | RNA-2 (GP) | PPSMV-I |
| 30 | 726 | RNA-1 (RdRp) | PPSMV-II |
| 35 | 631 | RNA-3 (NCP) | PPSMV-II |
| 37 | 598 | RNA-1 (RdRp) | PPSMV-II |
| 39 | 575 | RNA-3 (NCP) | PPSMV-II |
| 49 | 502 | RNA-1 (RdRp) | PPSMV-II |
| 57 | 419 | RNA-2 (GP) | PPSMV-I |
| 59 | 405 | RNA-1 (RdRp) | PPSMV-II |
| 61 | 394 | RNA-1 (RdRp) | PPSMV-II |
| 111 | 261 | RNA-3 (NCP) | PPSMV-I |
| 155 | 177 | RNA-3 (NCP) | PPSMV-I |

| **Contig number** | **Sequence length (bp)** | **Characters of reads/contigs**  **(Genomic segments)** | **Viral homology**  **(BlastX)** |
| --- | --- | --- | --- |
| **Bng-1** |  |  |  |
| 2 | 4638 | RNA-1 (RdRP) | PPSMV-II |
| 4 | 2211 | RNA-1 (RdRP) | PPSMV-II |
| 5 | 2064 | RNA-2 (GP) | PPSMV-II |
| 7 | 1831 | RNA-5 | PPSMV-II |
| 25 | 889 | RNA-4 (MP) | PPSMV-II |
| 58 | 524 | RNA-6 | PPSMV-II |
| 76 | 421 | RNA-4 (MP) | PPSMV-II |
| 98 | 364 | RNA-3 (NCP) | PPSMV-II |
| 215 | 238 | RNA-3 (NCP) | PPSMV-II |
| 229 | 316 | RNA-3 (NCP) | PPSMV-II |
| 243 | 220 | RNA-2 (GP) | PPSMV-II |
| 265 | 199 | RNA-3 (NCP) | PPSMV-II |
| 276 | 199 | RNA-4 (NCP) | PPSMV-II |
| 283 | 185 | RNA-3 (NCP) | PPSMV-II |
| 388 | 116 | RNA-3 (NCP) | PPSMV-II |
| 446 | 103 | RNA-3 (NCP) | PPSMV-II |

| P5 Protein (RNA-5) | PPSMV-1 (HF945448) | RNA-5*PPSMV-B Isolate | PPSMV-2(HG939489) | PPSMV-II* (LN898418) |
| --- | --- | --- | --- | --- |
| PPSMV-1 (HF945448) | 100.00 | 97.67 | 90.49 | 86.47 |
| PPSMV-II:RNA-5* PPSMV-B Isolate | 97.67 | 100.00 | 90.49 | 87.74 |
| PPSMV-2(HG939489) | 90.49 | 90.49 | 100.00 | 94.93 |
| PPSMV-II* (LN898418) | 86.47 | 87.74 | 94.93 | 100.00 |

**Supplementary Table 3:** Comparison of percent amino acid similarity of RNA-5 in PPSMVs

* Sequence characterized in this study

**Supplementary Table 4:** Sterility mosaic disease (SMD) affected pigeonpea collected from different locations in India, infected by distinct PPSMV isolates

----------------------------------------------------------------------------------------------------------------

Location/Cultivar Symptom PPSMV Isolate PPSMVs Samples

Detected

----------------------------------------------------------------------------------------------------------------

**Chevella (TS)** PPSMV-P sub-isolate

*Errakandulu* SM Chevella PPSMV-I Mg-1, Mg-2, Alr

ICP8863 SM and ICP8863*

ICP2376 RS PPSMV-II ICP2376*

**Bangaluru (KA)**

Un Known cultivar-1 SM PPSMV-B Isolate PPSMV-II Bng-1

Un Known cultivar-2 SM PPSMV-II Bng-2

Un Known cultivar-3 SM PPSMV-II Bng-3

**Coimbatore (TN)**

CO-5 SM PPSMV-C Isolate PPSMV-II CO-5

CO-6 SM PPSMV-II CO-6

ICP8863 SM PPSMV-II ICP8863

----------------------------------------------------------------------------------------------------------------

SMD samples Mg-1, Mg-2 and Alr collected from three pigeonpea fields in Chevella area (Telangana) SMD samples Bng-1 to Bng-3 collected from three pigeonpea fields in Bangaluru region (Karnataka)

Infected CO cultivars and pigeonpea cv. ICP8863 collected from TNAU research farm (Tamil Nadu)

SM = Systemic mosaic with sterility; RS= Ring spot symptoms with no sterility; * = Mite inoculated

pigeonpea cultivars. *Note*. Pigeonpea cultivars infected by PPSMV-P sub-isolate Chevella contain PPSMV-I and PPSMV-II.

**Supplementary Table 5:** List of primers used for genome amplification and sequencing of PPSMV-I and PPSMV-II

| **Primer region** | **Primer sequence (5’-3’)**  **(For PPSMV-I)** | **Targeted segment** |
| --- | --- | --- |
| RNA-1 FP (5’)* | AGTAGTGAGCTCCCTAAATAACAACTCAGTC | RNA-1 |
| 1055RP | TGTAGGTCTATATTTTCCAGTTTTATG | RNA-1 |
| 957FP | GCAAACACAATATTGCACGAAGATTG | RNA-1 |
| 2010RP | GATTCAATAACTTAAGTCTTTGCATGTT | RNA-1 |
| 1930FP | CAATCAATTACTGGGGATTCATCACCA | RNA-1 |
| 3140RP | GACTTTTGTATGCTTGCTTAACATTC | RNA-1 |
| 3024FP | AGTTACACAAAGCTTCTAGGTGAC | RNA-1 |
| 4202RP | CATATTATAAGTGGACATAGTTAGATGG | RNA-1 |
| 4091FP | GCATCTTATAGCGGTTATATAAATAATGC | RNA-1 |
| 5151RP | AATTAAGGATTTCATTCTTCTTATCAG | RNA-1 |
| 5058FP | CTAAAAGTGTCTATATGGAGGAGTATG | RNA-1 |
| 6264FP | CCTTTATATCATCAATACGTCCAAC | RNA-1 |
| 6166FP | ATTCATGGTTAATCAGTATAACAGGG | RNA-1 |
| RNA-1 RP (3’)`* | AGTAGTGTTCTCCCTAAATAACTAATACAAAG | RNA-1 |
| RNA-2 FP* | AGTAGTGAACTCCTCATAAACCAATCAAAC | RNA-2 |
| 1224RP | CAAAGCAGCCAATTGAGGGTC | RNA-2 |
| 1125FP | CAATACCATCTGTTGAGATTAGCA | RNA-2 |
| RNA-2 RP* | AGTAGTGTTCTCCTCATAAAACAAAAGCAA | RNA-2 |
| RNA-3 FP* | AGTAGTGAGCTCCCATAAATACAACAAG | RNA-3 |
| 610 FP | GGTCAGATCAGAACCTGAATGT | RNA-3 |
| RNA-3 RP* | AGTAGTGTTCTCCCATAAATAATAAAGC | RNA-3 |
| RNA-4 FP* | AGTAGTGAACTCCTTTACAATACTAAATC | RNA-4 |
| 647FP | GCTTCCTTGACATGTCTTGGA | RNA-4 |
| RNA-4 RP* | AGTAGTGTTCAGACGAAGTTGTTAATAAT | RNA-4 |
| RNA 5 FP* | AGTAGTGAGCTCCCATAATATGAGAAGTT | RNA-5 |
| 936RP | CATCATTGTATATAGCATTACTTATTTC | RNA-5 |
| 831FP | ATAACTTGATTTTCAGGAAAGATGATAT | RNA-5 |
| RNA5 RP* | AGTAGTGTTCTCCCATAATTGAAAACAAAATC | RNA-5 |

| **Primer region** | **Primer Sequence (5’- 3’)**  **(For PPSMV-II)** | **Targeted segment** |
| --- | --- | --- |
| RNA1FP* | AGTAGTGAACTCCCTTTAATACTATAAA | RNA-1 |
| 341-362 FP | AAGGCATGATGTACTACTTCTATCAA | RNA-1 |
| 760-780 FP | ATGCAAAGTGGACTAATATCTGAT | RNA-1 |
| 1050-1072 RP | TAGATGTTAAATTAGTGTTCTCTAAGC | RNA-1 |
| 1050-1072 FP | GCTTAGAGAACACTAATTTAACATCTA | RNA-1 |
| 2135- 21 59RP | CACAATATCATCTCTAAGTATAACTGA | RNA-1 |
| 3004 RP | CACCTTTAATGGTTTCTATGTATATTTCT | RNA-1 |
| 3694 RP | GATTACCTTGAAGCCAGTTACTTCTAA | RNA-1 |
| 3955- 3977 RP | ATAAAATGTACTGATATAAGTCTTC | RNA-1 |
| 4557-4584 FP | AAATCTATTATTAATGTTGTCAGTCTA | RNA-1 |
| 4557-4584 RP | TAGACTGACAACATTAATAATAGATTT | RNA-1 |
| 5999-6017 RP | CAGCACATTGAGTGAATGTCC | RNA-1 |
| 6732-6757 RP | ATCATTGTGACATGTTGTTGATAG | RNA-1 |
| RNA1 RP* | AGTAGTGTTCTCCCTTTAATTATAATGAC | RNA-1 |
| RNA2 FP* | AGTAGTGAACTCCTCTTAATACTGAAAAATC | RNA-2 |
| 1139 RP | GTACTCGCTTGATTCTAACATAAATGG | RNA-2 |
| 1032 FP | AGTAGTGTTCTCCTCTTAAAGAAAACAA | RNA-2 |
| RNA2 RP* | AGTAGTGTTCTCCTCTTAAAGAAAACAA | RNA-2 |
| RNA3 FP* | AGTAGTGAACTCCCATAATAACTAATCA | RNA-3 |
| 1189 RP | AGAGCCACAAGCTCTACCGATTG | RNA-3 |
| RNA-3RP* | AGTAGTGTTCTCCCATAATTGA | RNA-3 |
| RNA4 FP* | AGTAGTGAACTCCTTACAATACAATTAAC | RNA-4 |
| 100-122FP | ATGCCGACGATCGTTTCAATGAT | RNA-4 |
| (1308 RP) | ATGGTATCACTTATAATTAATTATC | RNA-4 |
| RNA4 RP* | AGTAGTGTTCTCCTTACAAGATAA | RNA-4 |
| RNA-5 FP* | AGTAGTGTTCTCCCATAATTGAA | RNA-5 |
| 1065 RP | GTAACAGCTTCAAAATTAGATATTATC | RNA-5 |
| 909 FP | AGGTTGGAGCTTTGAAATAAGTAATGC | RNA-5 |
| RNA-5 RP* | AGTAGTGTTCTCCCATAATTGAA | RNA-5 |
| RNA-6 FP* | AGTAGTGAACTCCCTATAACAAGA | RNA-6 |
| 890RP | TAGCTACGGTCATTGAAACATATCATAT | RNA-6 |
| RNA-6 RP* | AGTAGTGTTCTCCCTATAAACAAAAGT | RNA-6 |

FP: Forward Primer RP: Reverse Primer***:** Primers used for amplification; other primers were used for sequencing.

**Supplementary Table 6: Table showing details of number of reads and their quality analysis**

| **S.No.** | **Mg-1** | **Mg-2** | **Bng-1** |
| --- | --- | --- | --- |
| Total Number of Reads | 18530946 (18.53 millions) | 23759088 (23.76 millions) | 65949520 (65.95 millions) |
| Maximum Read Length | 36 | 36 | 36 |
| Minimum Read Length | 16 | 16 | 36 |
| Mean Read Length | 27 | 23 | 36 |
| Total Number of HQ Reads ^1*^ | 18101495 (18.10 millions) | 23374266 (23.37 millions) | 62333400 (62.33 millions) |
| Percentage of HQ Reads | 97.683% | 98.380% | 94.517% |
| Total Number of Bases | 502828301 bases | 553711483 bases | 2374182720 bases |
| Total Number of Bases in Mb | 502.8283 Mb | 553.7115 Mb | 2374.18272 Mb |
| Total Number of HQ Bases ^2*^ | 491833757 bases | 543772933 bases | 2256307380 bases |
| Total Number of HQ Bases in Mb | 491.8338 Mb | 543.7729 Mb | 2256.30738 Mb |
| Percentage of HQ Bases | 97.813% | 98.205% | 95.035% |
| Total Number of Non-ATGC Characters | 39690 bases | 53688 bases | 455297 bases |
| Total Number of Non-ATGC Characters in Mb | 0.040 Mb | 0.054 Mb | 0.455 Mb |
| Percentage of Non-ATGC Characters | 0.008% | 0.010% | 0.019% |
| Number of Reads with Non-ATGC Characters | 39676 | 52882 | 312781 |
| Percentage of Reads with Non-ATGC Characters | 0.214% | 0.223% | 0.474% |
| Contigs generated | 229 | 229 | 461 |
| Maximum contig length | 5822 | 2111 | 5409 |

^1*^ >70% of bases in a read with >20 phred score and reads which are of low quality can be trimmed and used

^2*^ bases with >20 phred score
